# The ratio of systolic and diastolic pressure is associated with carotid and femoral atherosclerosis

**DOI:** 10.3389/fcvm.2024.1353945

**Published:** 2024-03-08

**Authors:** Yuanping Wang, Cheng Chen, Qiaofen Lin, Qingling Su, Yiquan Dai, Hongyu Chen, Tianmin He, Xiantao Li, Ruimei Feng, Wuqing Huang, Zhijian Hu, Jun Chen, Shanshan Du, Pingfan Guo, Weimin Ye

**Affiliations:** ^1^ Department of Epidemiology and Health Statistics, School of Public Health, Fujian Medical University, Fuzhou, China; ^2^ Department of Vascular Surgery, The First Affiliated Hospital, Fujian Medical University, Fuzhou, China; ^3^ Department of Epidemiology, School of Public Health, Shanxi Medical University, Taiyuan, Shanxi, China; ^4^ Department of Medical Epidemiology and Biostatistics, Karolinska Institutet, Stockholm, Sweden

**Keywords:** SDR, carotid, femoral, atherosclerosis, hypertension

## Abstract

**Background:**

Although the impact of hypertension on carotid intima-media thickness (IMT) and plaques has been well established, its association with femoral IMT and plaques has not been extensively examined. In addition, the role of the ratio of systolic and diastolic pressure (SDR) in the subclinical atherosclerosis (AS) risk remains unknown. We assessed the relationship between SDR and carotid and femoral AS in a general population.

**Methods:**

A total of 7,263 participants aged 35–74 years enrolled from January 2019 to June 2021 in a southeast region of China were included in a cross-sectional study. Systolic and diastolic blood pressure (SBP and DBP) were used to define SDR. Ultrasonography was applied to assess the AS, including thickened IMT (TIMT) and plaque in the carotid and femoral arteries. Logistic regression and restricted cubic spline (RCS) models were the main approaches.

**Results:**

The prevalence of TIMT, plaque, and AS were 17.3%, 12.4%, and 22.7% in the carotid artery; 15.2%, 10.7%, and 19.5% in the femoral artery; and 23.8%, 17.9% and 30.0% in either the carotid or femoral artery, respectively. Multivariable logistic regression analysis found a significant positive association between high-tertile SDR and the higher risk of overall TIMT (OR = 1.28, 95% CI = 1.10–1.49), plaques (OR = 1.36, 95%CI = 1.16–1.61), or AS (OR = 1.36, 95% CI = 1.17–1.57), especially in the carotid artery. RCS analysis further revealed the observed positive associations were linear. Further analyses showed that as compared to the low-tertile SDR and non-hypertension group, high-tertile SDR was associated with increased risks of overall and carotid TIMT, plaques, or AS in both groups with or without hypertension.

**Conclusions:**

SDR is related to a higher risk of subclinical AS, regardless of hypertension or not, suggesting that as a readily obtainable index, SDR can contribute to providing additional predictive value for AS.

## 1 Introduction

Peripheral artery disease (PAD) is an atherosclerotic disease of the arteries, which is always underappreciated due to being symptomless in its early stages. As a predecessor of cardiovascular diseases (CVD), it confers an elevated risk of various adverse outcomes. It has been recognized as an increasing global public health issue, receiving more and more attention in recent years (1–3). It is known that the intima of middle- and large-sized arteries are most vulnerable to atherosclerosis (AS), especially the sites of vessel branching, due to the nature of the blood flow (4). Thus, AS screening in carotid and femoral arteries is of great importance to CVD prevention, especially in the general population. However, early AS detection in the general population has mainly focused on the carotid artery (3), while femoral AS is still underrecognized (2).

Hypertension is defined by systolic and diastolic blood pressure (SBP and DBP), referring to the pressure produced by arteries during systolic and diastolic activities of the heart, which could reflect the early hemodynamic changes, perfusion of organs, and heart systolic function. Lower SBP/DBP levels have been strictly recommended to decrease CVD risk (5–7). It can also greatly reduce the risk of atherosclerotic CVD and death (8). However, there is a dearth of studies distinguishing the role of BP in the AS across different sites. In addition to the absolute value of SBP or DBP, recent studies proposed a novel index, the ratio of SBP and DBP (SDR) (9). Some studies have shown that hypertensive subjects with proper SDR may suffer from less severe forms of end-organ damage than those with ratios significantly deviating from the mean ratio (10), indicating the potentially additional predictive value of SDR in cardiovascular outcomes. However, its implication in AS risk has previously not been evaluated.

In the current study, we conducted a cross-sectional study in southeastern China to explore the association between SDR and AS in arteries, including carotid and femoral arteries.

## 2 Material and methods

### 2.1 Study design and participants

Data for this cross-sectional study were collected from the baseline survey of the Fuqing Cohort Study (11–13), conducted in Fuqing City, Fujian Province, China. Native residents aged 35–74 years were recruited, and a total of 10,193 residents participated in the baseline survey of the cohort from January 2019 to June 2021. Participants were excluded from the analyses if they met the criteria below:

1. individuals aged <35 or ≥75 years (*n* = 88);
2. incomplete questionnaire survey on disease history (*n* = 591);
3. missing data on BP measurements (*n* = 151);
4. a self-reported history of coronary heart disease, stroke, or malignant tumor (*n* = 19);
5. without measurement of peripheral AS (*n* = 2,081).

After excluding 2,930 participants, a total of 7,263 individuals were included in the final study. Permission for the cohort study was obtained from the Ethics Committee of Fujian Medical University (approval number: [2017–07] and [2020–58]) before data collection. Written informed consent was obtained from all the participants prior to the enrollment of this study.

### 2.2 Data collection and variables

The details of data collection have been described in previous studies (13, 14). Briefly, a structured questionnaire compiled by the Fujian Cohort Research Center was used to collect information on demographic and sociological characteristics (age, gender, occupation, and education), disease and medication history, family history of disease, and lifestyles (smoking, drinking, and physical activity). Anthropometric measurements were taken to determine height (cm) and weight (kg), and body mass index (BMI) was calculated and classified into underweight, normal weight, overweight, and obese groups according to the recommendations of the Working Group on Obesity in China (15). Fasting venous blood was taken to determine fasting blood glucose (FBG), glucose hemoglobin A1c (HbA1c), total cholesterol, triglyceride, high density lipoprotein cholesterol and low density lipoprotein cholesterol. Diabetes refers to a measured FBG ≥ 7.0 mmol/L or HbA1c ≥ 6.5%, self-reported history of diabetes, and/or taking antidiabetic drugs. Hyperlipidemia was defined as present if ≥1 of the following criteria are satisfied: total cholesterol >6.2 mmol/L, triglyceride >2.3 mmol/L, high-density lipoprotein cholesterol <1.0 mmol/L, or low-density lipoprotein cholesterol >4.1 mmol/L. Treatment of AS was referred to using drugs that could act on the vascular wall.

### 2.3 Assessment of exposures

SDR was defined as the ratio between SBP and DBP. BP measurements were taken on the right upper arm at the heart level by trained employees using an electronic blood pressure monitor (OMRON, U30, Japan). SBP and DBP were recorded twice. A third measurement was taken if there was a difference of more than 5 mmHg between the two measurements. The average of the two closest measurements was used to define the value of BP.

Hypertension was referred to SBP ≥ 140 mmHg or/and DBP ≥ 90 mmHg, self-reported history of hypertension (HT), or taking antihypertensive drugs. Normotension was defined as averaged SBP ranging from 90 to 119, or/and DBP ranging from 60 to 79 mmHg without self-reported diagnosis or treatment of HT; prehypertension (PreHT) was defined as averaged SBP ranging from 120 to 139, or/and DBP ranging from 80 to 89 mmHg without self-reported diagnosis or treatment of HT.

### 2.4 Assessment of outcomes

AS was the primary outcome, defined as the presence of thickened intima-media thickness (TIMT) or/and plaques in the carotid or/and femoral arteries. TIMT and plaques in the carotid or/and femoral arteries were the secondary outcomes. B-mode ultrasound imaging (EDGEII, Sono Sound, America) was performed to determine IMT and plaques in the carotid and femoral arteries synchronously by qualified surgeons, as previously reported (11, 12).

Briefly, TIMT was reported if there exists an IMT ≥ 1.0 mm at any one of the four arteries. IMT ≥1.5 mm, or a focal structure encroaching into the arterial lumen by at least 0.5 mm, or >50% of the surrounding IMT value was regarded as the presence of plaque (16). According to the specific vascular location of TIMT, plaques, and AS, participants were diagnosed as only carotid TIMT, plaques, and AS (C-TIMT, C-P, and C-AS), only femoral TIMT, plaques, and AS (F-TIMT, F-P, and F-AS), and either carotid or femoral TIMT, plaques, and AS (CF-TIMT, CF-P, and CF-AS).

### 2.5 Statistical analysis

All statistical analyses were performed using SAS 9.4 statistical software, and a *P*-value < 0.05 was considered statistically significant. Individuals were divided into low-, medium-, and high-SDR groups according to their tertiles. Continuous variables were expressed as mean and standard deviations (SD), and compared using one-way ANOVA among three groups. Categorical variables were reported as numbers and proportions, and compared using the Chi-squared tests across groups. The correlation coefficients between SDR and SBP or DBP were determined using the Spearman correlation test and displayed with a scatter plot. The joint effect of SBP and DBP on AS risk was first clarified by univariable logistic regression, the estimates of which were shown as heat maps. Univariable and multivariable logistic regression analyses were used to generate odds ratios (ORs) and 95% confidential intervals (CIs) for the association between SDR groups and TIMT, plaques, or AS in carotid, femoral, or both arteries, respectively. Multivariable models included age- and sex-adjusted models and a fully-adjusted model, and the fully-adjusted model included age, sex, BMI, occupation, education, alcohol drinking, smoking, diabetes, dyslipidemia, SBP, and treatment of AS. A restricted cubic spline (RCS) plot with five knots was used to present the linear or nonlinear relationship between continuous SDR level and AS risk.

Considering the joint effects of HT and SDR on AS risk, we regrouped all participants into six subgroups according to SDR and HT, and they were low-SDR and non-HT (L-SDR & non-HT), medium-SDR and non-HT (M-SDR & non-HT), high-SDR and non-HT (H-SDR & non-HT), low-SDR and HT (L-SDR & HT), medium-SDR and HT (M-SDR & HT), and high-SDR and HT (H-SDR & HT). Multivariable adjusted logistic regression analyses were used to estimate the ORs of joint effects of SDR and HT for AS risk, with L-SDR & non-HT group as the reference.

The ORs of preHT and HT (normotension, PreHT, and HT) for AS risk were also calculated in fully adjusted logistic regression analyses. The absolute SBP value was categorized into <110 mmHg, 110 mmHg–119 mmHg, 120 mmHg–124 mmHg, 125 mmHg–129 mmHg, 130 mmHg–139 mmHg, and ≥140 mmHg, while DBP was categorized into <75 mmHg, 75 mmHg–79 mmHg, 80 mmHg–84 mmHg, 85 mmHg–89 mmHg, and ≥90 mmHg. A heat map from GraphPad Prism was applied to display the ORs between each BP subgroup and AS risk. Considering the significant differences in BP and SDR levels between hypertensive and non-hypertensive populations, we performed a stratified analysis. The relationship between SDR and AS risk in these two groups was discussed respectively. In addition, we also conducted an RCS analysis of the risk of SDR and AS to explore the potential linear and non-linear association between them, and to explore whether the potential optimal SDR for non-hypertensive and hypertensive populations was similar or not.

To further explore the association between SDR and AS risk, we also performed a sensitivity analysis. Participants with antihypertensive medicine were excluded, and the association between SDR, HT, and their six subgroups and AS risk were reanalyzed, considering the potential effect of the medicine on BP values and AS risk.

## 3 Results

### 3.1 The demographic characteristics of the study population

**Figure 1:**
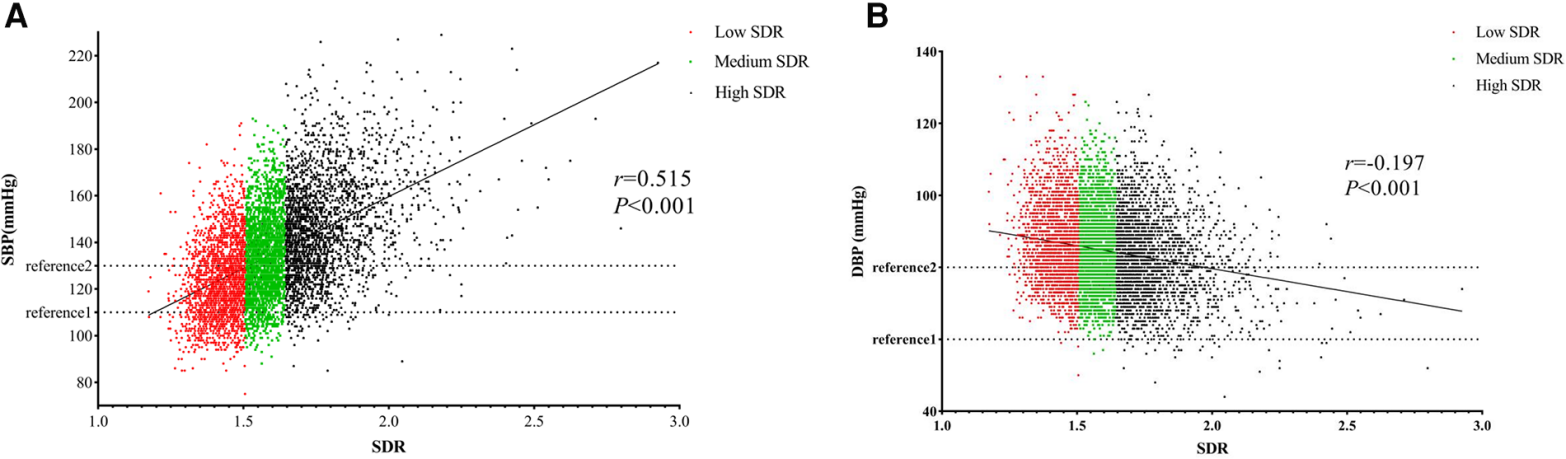
Distribution of SDR with SBP (**A**) and DBP (**B**) SDR, the ratio of systolic and diastolic pressure; SBP, systolic blood pressure; DBP, diastolic blood pressure.

The average SBP and DBP of 7,263 participants were 134.41 (±20.71) mmHg and 84.55 (±11.22) mmHg, respectively. The prevalence of HT was 46.3%. The SDR ranged from 1.17 to 2.93 (Mean: 1.59, SD: 0.17). According to its tertiles, low-, medium-, and high-SDR groups were defined as SDR < 1.506, 1.506–1.643, and > 1.643 (Table 1). The SDP, DBP, and HT prevalence rates were significantly higher in high-SDR groups. Participants with high SDR were more likely to be older, female, obese, receiving treatment for AS, less educated, more likely to be farmers or unemployed, less likely to be current smokers or alcohol drinkers, and have diabetes and dyslipidemia. The distribution of SBP and DBP across SDR is depicted in Figure 1. The scatter plots reveal a notable positive correlation between SDR and SBP, and a moderate inverse correlation between SDR and DBP. The correlation coefficients between SDR-SBP and SDR-DBP were 0.515 and −0.197, respectively.

**Table 1:** Characteristics of the study population by systolic/diastolic ratio (SDR) of blood pressure.

Variable	Overall	Low SDR	Medium SDR	High SDR	*P*
	(*N* = 7,263)	(<1.506 (*N* = 2,422)	(1.506–1.643 (*N* = 2,417)	(>1.643 (*N* = 2,424)	
SDR	1.59 ± 0.17	1.43 ± 0.06	1.57 ± 0.04	1.79 ± 0.14	***<0***.***001***
Blood pressure, mmHg					
SBP	134.41 ± 20.71	123.35 ± 15.56	133.06 ± 17.29	146.80 ± 21.61	***<0***.***001***
DBP	84.55 ± 11.22	86.59 ± 10.80	84.71 ± 10.79	82.36 ± 11.66	
Hypertension	3,364 (46.3)	899 (37.1)	928 (38.4)	1,537 (63.4)	***<0***.***001***
Age, years	57.48 ± 9.80	53.85 ± 9.53	56.95 ± 9.52	61.66 ± 8.71	***<0***.***001***
Male	2,578 (35.5)	1,009 (41.7)	847 (35.0)	722 (29.8)	***<0.001***
BMI, kg/m^2^	24.10 ± 3.33	23.89 ± 3.34	24.13 ± 3.41	24.29 ± 3.23	***<0.001***
Education level					
Illiterate	2,713 (37.4)	661 (27.3)	876 (36.2)	1,176 (48.5)	***<0***.***001***
Primary school	2,394 (33.0)	800 (33.0)	865 (35.8)	729 (30.1)	
Middle school	1,567 (21.6)	674 (27.8)	503 (20.8)	390 (16.1)	
High school and above	589 (8.1)	287 (11.9)	173 (7.2)	129 (5.3)	
Occupation					
Farmer/unemployment	5,253 (72.3)	1,568 (64.7)	1,730 (71.6)	1,955 (80.7)	***<0***.***001***
Worker	772 (10.6)	303 (12.5)	289 (12.0)	180 (7.4)	
Sales/service	428 (5.9)	197 (8.1)	135 (5.6)	96 (4.0)	
White collar	712 (9.8)	315 (13.0)	224 (9.3)	173 (7.1)	
Other	98 (1.3)	39 (1.6)	39 (1.6)	20 (0.8)	
Smoking status					
Never	5,354 (73.7)	1,659 (68.5)	1,793 (74.2)	1,902 (78.5)	***<0***.***001***
Former	630 (8.7)	228 (9.4)	197 (8.2)	205 (8.5)	
Current	1,279 (17.6)	535 (22.1)	427 (17.7)	317 (13.1)	
Alcohol drinking status					
Never	6,393 (88.0)	2,083 (86.0)	2,130 (88.1)	2,180 (89.9)	***<0***.***001***
Former	268 (3.7)	113 (4.7)	90 (3.7)	65 (2.7)	
Current	602 (8.3)	226 (9.3)	197 (8.2)	179 (7.4)	
Diabetes	1,198 (16.5)	290 (12.0)	330 (13.7)	578 (23.8)	***<0***.***001***
Dyslipidemia	2,840 (39.1)	848 (35.0)	937 (38.8)	1,055 (43.5)	***<0***.***001***
Treatment of AS	270 (3.7)	73 (3.0)	79 (3.3)	118 (4.9)	***<0***.***05***
Carotid artery					
TIMT	1,256 (17.3)	306 (12.6)	380 (15.7)	570 (23.5)	***<0***.***001***
Plaque	904 (12.4)	221 (9.1)	255 (10.6)	428 (17.7)	
AS	1,648 (22.7)	397 (16.4)	502 (20.8)	749 (30.9)	
Femoral artery					
TIMT	1,102 (15.2)	309 (12.8)	334 (13.8)	459 (18.9)	***<0***.***001***
Plaque	775 (10.7)	207 (8.6)	242 (10.0)	326 (13.5)	
AS	1,417 (19.5)	389 (16.1)	442 (18.3)	586 (24.2)	
Carotid artery or femoral artery					
TIMT	1,729 (23.8)	458 (18.9)	526 (21.8)	745 (30.7)	***<0***.***001***
Plaque	1,297 (17.9)	329 (13.6)	389 (16.1)	579 (23.9)	
AS	2,178 (30.0)	571 (23.6)	674 (27.9)	933 (38.5)	

Data are shown as *n* (%) for categorical variables and mean ± standard deviation for continuous variables.

SBP, systolic blood pressure; DBP, diastolic blood pressure; BMI, body mass index; TIMT, thickened intima-media thickness; AS, atherosclerosis.

The bold italics values represent statistically significant between groups.

In the whole population, the prevalence rates of TIMT, plaques, and AS were 17.3%, 12.4%, and 22.7% in the carotid artery, and 15.2%, 10.7%, and 19.5% in the femoral artery, respectively. When defining AS in either carotid or femoral artery, the prevalence rates of TIMT, plaques, and AS were 23.8%, 17.9%, and 30.0%. From the low- to high-SDR group, the prevalence of TIMT, plaques, and AS in carotid and/or femoral arteries all increased gradually.

### 3.2 The effects of SBP and DBP on AS

**Figure 2:**
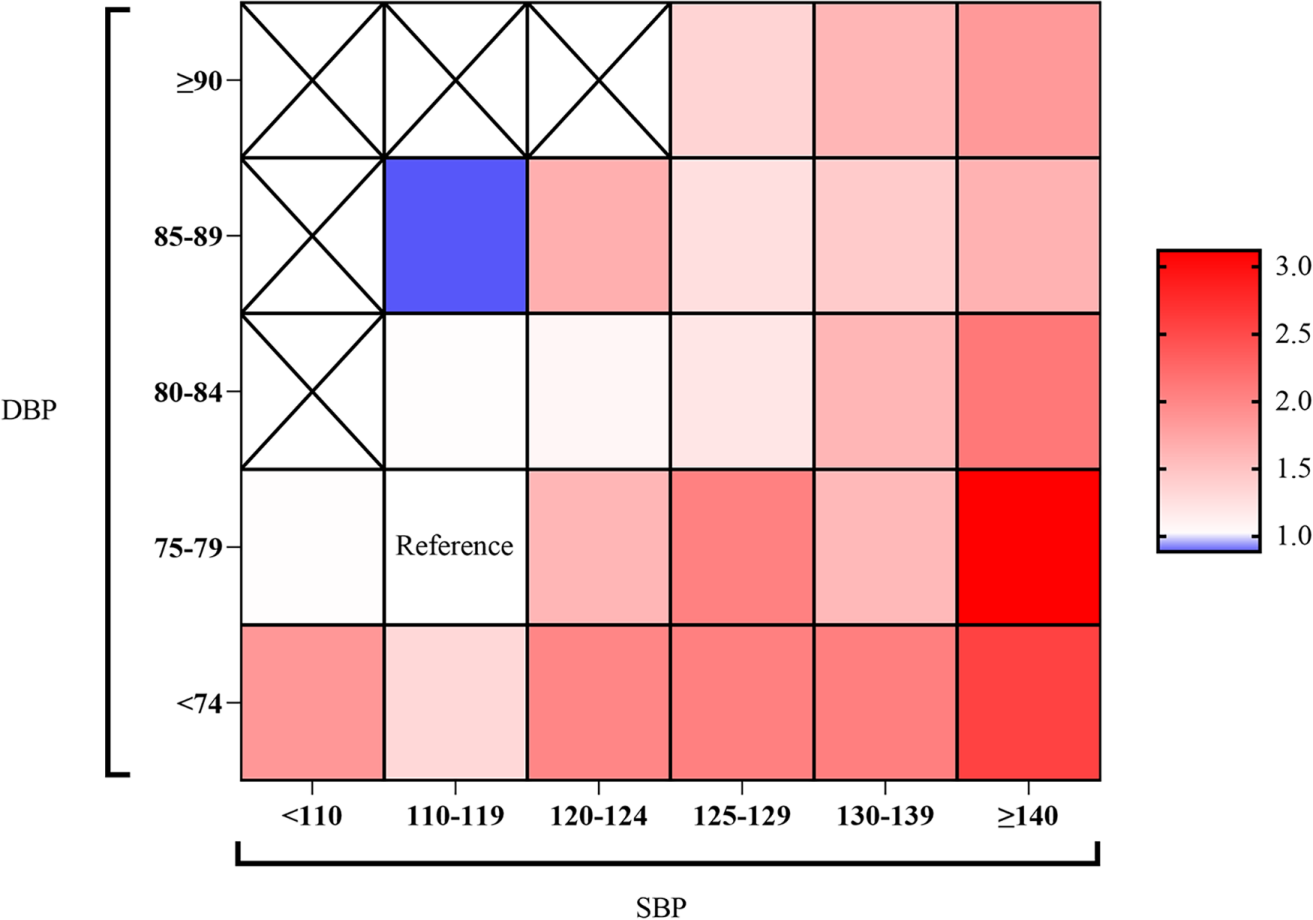
Heat map of the association between BP levels and carotid and femoral AS risk. SBP level was at the x-axis, and DBP level was at the y-axis. The color represents the odds ratio of AS risk at a given SBP/DBP level. SBP/DBP at 110–119/75–79 mmHg was set as a reference group. The blanks with “×” indicated missing OR values due to the small sample size.

The heatmap of the ORs in Figure 2 shows the joint effects of SBP and DBP on CF-AS risk, in which the blank in white was the reference group (SBP 110–119 mmHg and DBP 75–79 mmHg), the red blank marked an OR > 1.0, the blue marked an OR < 1.0, and the OR became higher as the color deepened. Overall, with the increasing SBP, the ORs for AS became higher, but the values at a given SBP group varied by DBP groups. As compared to the reference, both lower and higher DBP seemed to be associated with a higher prevalence of AS. These findings suggested that an index combining SBP and DBP, such as SDR, might provide more information.

### 3.3 The association between SDR and AS

The low-SDR group was set as a reference in logistic regression models. As shown in Table 2, after adjusting for covariates, the ORs (95% CIs) of high-SDR for C-TIMT, and C-AS were significant, with values of 1.39 (1.15,1.68), and 1.38 (1.16,1.63), respectively. On the contrary, the ORs of medium-SDR for C-TIMT, C-P, and C-AS were all non-significant. In the femoral artery, neither medium- nor high-SDR was associated with F-TIMT, F-P, and F-AS risk. Combining the carotid and femoral arteries, a significantly higher risk for CF-TIMT, CF-AS could be observed in high-SDR, and the ORs (95%CI) were 1.29 (1.09,1.53), and 1.26 (1.07,1.48), respectively. The associations between medium-SDR and CF-TIMT, CF-P, and CF-AS were not significant.

**Table 2:** ORs and 95% CIs for prevalence of atherosclerosis subgroup by the systolic/diastolic ratio (SDR) of blood pressure.

Variables	Low SDR		Medium SDR		High SDR	
	*N* (%)	OR (95% CI)	*N* (%)	OR (95% CI)	*N* (%)	OR (95% CI)
Carotid artery						
TIMT	306 (12.6)		380 (15.7)		570 (23.5)	
Model 1		Reference		1.29 (1.10, 1.52)		2.13 (1.83, 2.48)
Model 2		Reference		1.13 (0.95, 1.34)		1.50 (1.26, 1.78)
Model 3		Reference		1.14 (0.95, 1.35)		1.50 (1.26, 1.77)
Model 4		Reference		1.10 (0.92, 1.31)		1.39 (1.15, 1.68)
Plaque	221 (9.1)		255 (10.6)		428 (17.7)	
Model 1		Reference		1.18 (0.97, 1.42)		2.14 (1.80, 2.54)
Model 2		Reference		1.01 (0.83, 1.23)		1.48 (1.22, 1.78)
Model 3		Reference		1.03 (0.84, 1.25)		1.47 (1.21, 1.77)
Model 4		Reference		0.92 (0.75, 1.13)		1.19 (0.97, 1.47)
AS	397 (16.4)		502 (20.8)		749 (30.9)	
Model 1		Reference		1.34 (1.16, 1.55)		2.28 (1.99, 2.62)
Model 2		Reference		1.16 (0.99, 1.36)		1.57 (1.34, 1.83)
Model 3		Reference		1.17 (0.99, 1.37)		1.56 (1.33, 1.82)
Model 4		Reference		1.10 (0.94, 1.30)		1.38 (1.16, 1.63)
Femoral artery						
TIMT	309 (12.8)		334 (13.8)		459 (18.9)	
Model 1		Reference		1.10 (0.93, 1.30)		1.60 (1.37, 1.87)
Model 2		Reference		0.95 (0.80, 1.14)		1.12 (0.95, 1.34)
Model 3		Reference		0.96 (0.80, 1.14)		1.13 (0.95, 1.35)
Model 4		Reference		1.01 (0.84, 1.21)		1.16 (0.96, 1.41)
Plaque	207 (8.6)		242 (10.0)		326 (13.5)	
Model 1		Reference		1.19 (0.98, 1.45)		1.66 (1.38, 2.00)
Model 2		Reference		1.04 (0.85, 1.28)		1.15 (0.94, 1.41)
Model 3		Reference		1.08 (0.88, 1.33)		1.18 (0.96, 1.44)
Model 4		Reference		0.99 (0.80, 1.23)		0.95 (0.76, 1.19)
AS	389 (16.1)		442 (18.3)		586 (24.2)	
Model 1		Reference		1.17 (1.01, 1.36)		1.67 (1.44, 1.92)
Model 2		Reference		1.02 (0.87, 1.20)		1.16 (0.99, 1.36)
Model 3		Reference		1.04 (0.88, 1.22)		1.17 (0.99, 1.38)
Model 4		Reference		1.04 (0.88, 1.23)		1.11 (0.93, 1.32)
Carotid artery or femoral artery						
TIMT	458 (18.9)		526 (21.8)		745 (30.7)	
Model 1		Reference		1.19 (1.04, 1.37)		1.90 (1.67, 2.17)
Model 2		Reference		1.02 (0.88, 1.19)		1.29 (1.11, 1.51)
Model 3		Reference		1.03 (0.88, 1.20)		1.30 (1.11, 1.51)
Model 4		Reference		1.05 (0.90, 1.23)		1.29 (1.09, 1.53)
Plaque	329 (13.6)		389 (16.1)		579 (23.9)	
Model 1		Reference		1.22 (1.04, 1.43)		2.00 (1.72, 2.32)
Model 2		Reference		1.06 (0.89, 1.25)		1.38 (1.17, 1.63)
Model 3		Reference		1.09 (0.92, 1.29)		1.39 (1.18, 1.65)
Model 4		Reference		0.98 (0.83, 1.17)		1.11 (0.93, 1.34)
AS	571 (23.6)		674 (27.9)		933 (38.5)	
Model 1		Reference		1.25 (1.10, 1.43)		2.03 (1.79, 2.30)
Model 2		Reference		1.08 (0.94, 1.25)		1.38 (1.19, 1.60)
Model 3		Reference		1.09 (0.95, 1.26)		1.38 (1.19, 1.59)
Model 4		Reference		1.07 (0.92, 1.24)		1.26 (1.07, 1.48)

TIMT, thickened intima-media thickness; AS, atherosclerosis; CI, confidence interval; OR, odds ratio; SDR, the ratio of systolic and diastolic blood pressure.

Model 1 was crude; Model 2 was adjusted for age and sex; Model 3 was adjusted for age, sex, BMI, occupation, education, alcohol drinking, smoking, diabetes, and dyslipidemia; Model 4 was adjusted for age, sex, BMI, occupation, education, alcohol drinking, smoking, diabetes, dyslipidemia, SBP, and treatment of AS.

**Figure 3:**
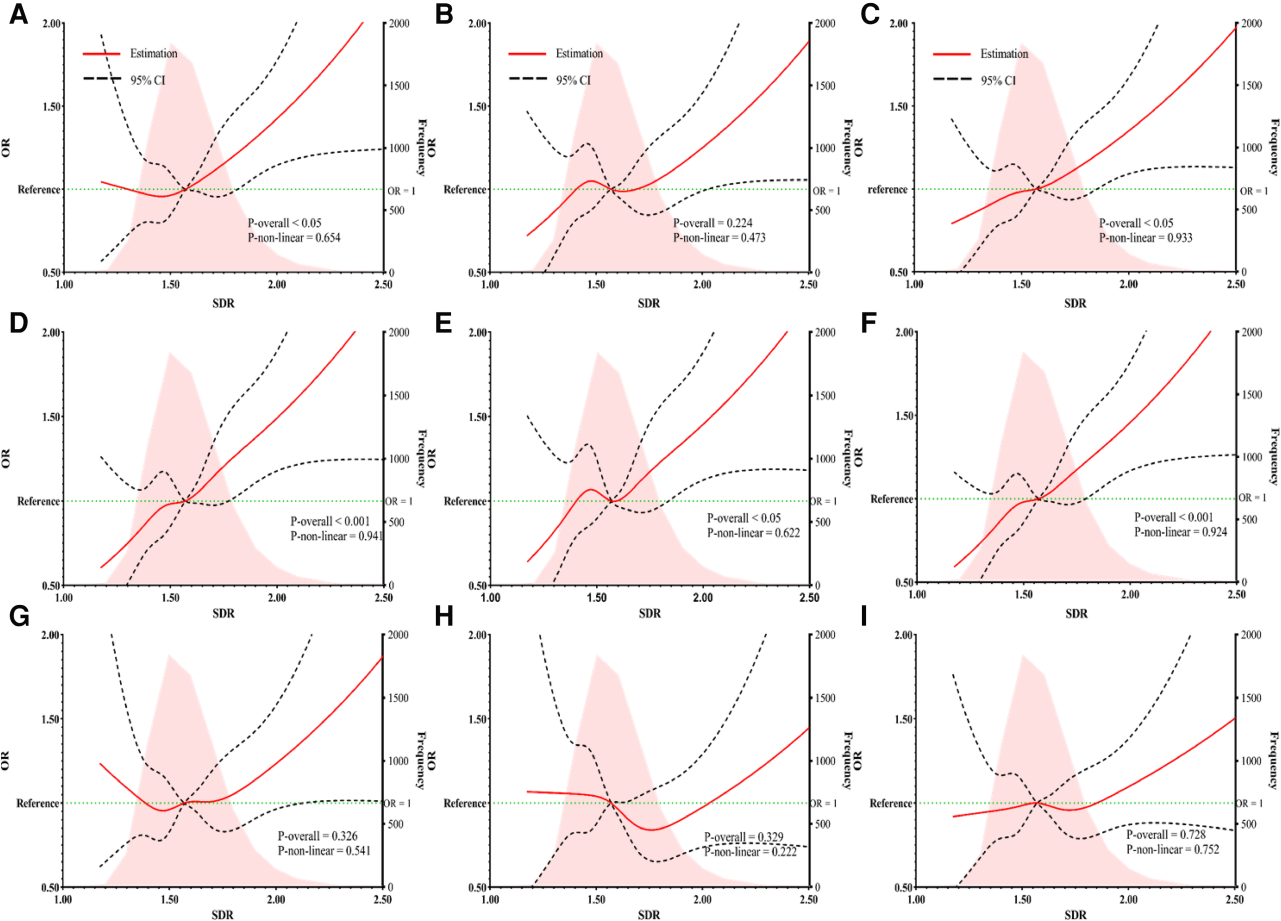
The RCS regression between SDR and the risk of carotid or femoral TIMT (**A**), plaque (**B**), and AS (**C**) the RCS regression between SDR and the risk of carotid TIMT (**D**), plaque (**E**), and AS (**F**) the RCS regression between SDR and the risk of femoral TIMT (**G**), plaque (**H**), and AS (**I**). SDR, the ratio of systolic and diastolic blood pressure; OR, odds ratio; CI, confidence interval.

Results from RCS regression are shown in Figure 3. Positive linear relationships were observed between SDR and CF-TIMT, CF-AS, C-TIMT, C-P, and C-AS.

### 3.4 The joint effects of SDR and HT on AS risk

**Figure 4:**
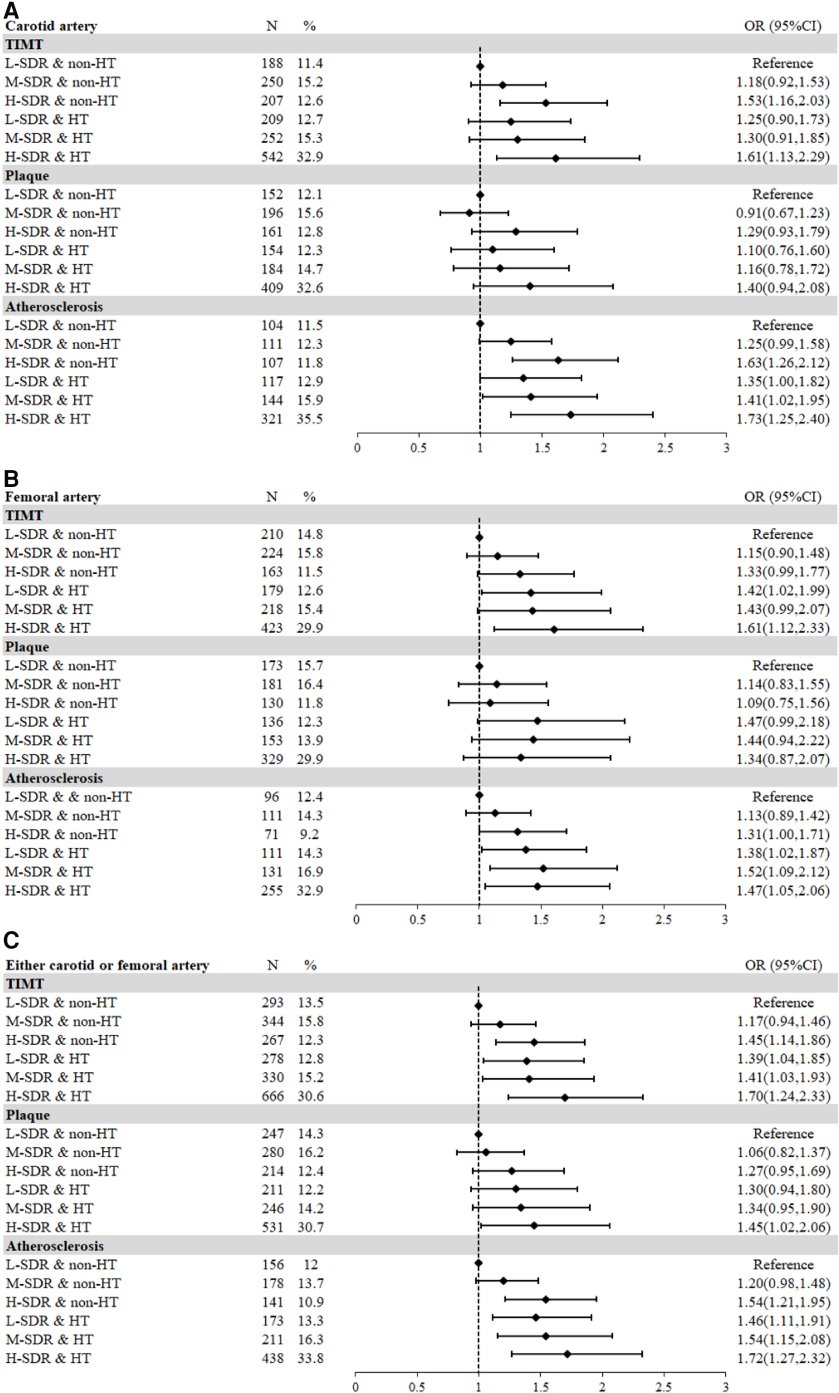
The joint effects of SDR and hypertension on AS risk. (**A**) Carotid artery; (**B**) Femoral artery; (**C**) Carotid or femoral arteries. TIMT, thickened intima-media thickness; AS, atherosclerosis; SDR, the ratio of systolic and diastolic blood pressure, OR, odds ratio; CI, confidence interval.

As compared with normotension, HT was only positively associated with F-P in fully adjusted logistic regression models (Table 3). Thus, we then explored the joint effects of SDR and HT on AS risk. Taking the group with L-SDR and non-HT as the reference, the ORs of M-SDR and non-HT, H-SDR and non-HT, L-SDR and HT, M-SDR and HT, and H-SDR and HT for AS risk were calculated from fully adjusted logistic regression models (Supplementary Table S1), and shown in forest plots (Figure 4). For carotid artery, H-SDR and non-HT, and H-SDR and HT were associated with higher C-TIMT risk, and the ORs of H-SDR and non-HT, L-SDR and HT, M-SDR and HT, and H-SDR and HT for C-AS were all significant and higher than 1.0, suggesting the additional predictive value of SDR for carotid AS in both HT and non-HT population (Figure 4A). For femoral artery, L-SDR and HT, and H-SDR and HT were associated with higher F-TIMT risk, and H-SDR and non-HT, L-SDR and HT, M-SDR and HT, and H-SDR and HT were associated with higher F-AS risk, suggesting that although no association of SDR with femoral AS in overall population, SDR could provide more predictive value for femoral AS in HT population (Figure 4B). In either carotid or femoral artery, H-SDR and non-HT, L-SDR and HT, M-SDR and HT, and H-SDR and HT were associated with CF-TIMT and CF-AS risk (Figure 4C).

**Table 3:** The association between prehypertension, hypertension, and AS risk..

Variables	Normotension		Prehypertension		Hypertension	
	*N* (%)	OR (95% CI)	*N* (%)	OR (95% CI)	*N* (%)	OR (95% CI)
Carotid artery						
TIMT	139 (9.7)		370 (15.0)		747 (22.2)	
Model 1		Reference		1.64 (1.33, 2.01)		2.65 (2.19, 3.22)
Model 2		Reference		1.23 (0.99, 1.52)		1.46 (1.19, 1.79)
Model 3		Reference		1.23 (0.98, 1.53)		1.41 (1.14, 1.75)
Model 4		Reference		1.03 (0.72, 1.48)		1.09 (0.72, 1.65)
Plaque	87 (6.1)		235 (9.5)		582 (17.3)	
Model 1		Reference		1.62 (1.26, 2.10)		3.23 (2.55, 4.08)
Model 2		Reference		1.23 (0.95, 1.60)		1.86 (1.45, 2.37)
Model 3		Reference		1.25 (0.96, 1.64)		1.87 (1.45, 2.40)
Model 4		Reference		1.10 (0.70, 1.71)		1.22 (0.73, 2.02)
AS	174 (12.2)		471 (19.1)		1,003 (29.8)	
Model 1		Reference		1.70 (1.41, 2.05)		3.07 (2.57, 3.65)
Model 2		Reference		1.27 (1.04, 1.55)		1.68 (1.39, 2.03)
Model 3		Reference		1.29 (1.05, 1.58)		1.65 (1.35, 2.01)
Model 4		Reference		1.12 (0.80, 1.57)		1.21 (0.82, 1.79)
Femoral artery						
TIMT	174 (12.2)		310 (12.6)		618 (18.4)	
Model 1		Reference		1.04 (0.85, 1.26)		1.63 (1.36, 1.95)
Model 2		Reference		0.76 (0.62, 0.93)		0.89 (0.73, 1.08)
Model 3		Reference		0.78 (0.63, 0.96)		0.93 (0.76, 1.14)
Model 4		Reference		1.11 (0.78, 1.58)		1.37 (0.90, 2.09)
Plaque	81 (5.7)		197 (8.0)		497 (14.8)	
Model 1		Reference		1.44 (1.11, 1.89)		2.89 (2.26, 3.68)
Model 2		Reference		1.09 (0.83, 1.44)		1.68 (1.30, 2.17)
Model 3		Reference		1.15 (0.87, 1.52)		1.75 (1.34, 2.28)
Model 4		Reference		1.34 (0.84, 2.13)		1.76 (1.03, 3.01)
AS	201 (14.1)		396 (16.0)		820 (24.4)	
Model 1		Reference		1.17 (0.97, 1.40)		1.97 (1.67, 2.33)
Model 2		Reference		0.86 (0.71, 1.04)		1.09 (0.91, 1.31)
Model 3		Reference		0.88 (0.72, 1.08)		1.13 (0.93, 1.37)
Model 4		Reference		1.12 (0.80, 1.56)		1.37 (0.93, 2.03)
Carotid artery or femoral artery						
TIMT	235 (16.4)		506 (20.5)		988 (29.4)	
Model 1		Reference		1.31 (1.11, 1.56)		2.12 (1.81, 2.48)
Model 2		Reference		0.95 (0.79, 1.14)		1.11 (0.93, 1.32)
Model 3		Reference		0.96 (0.79, 1.16)		1.11 (0.92, 1.33)
Model 4		Reference		1.08 (0.79, 1.47)		1.28 (0.88, 1.84)
Plaque	136 (9.5)		339 (13.7)		822 (24.4)	
Model 1		Reference		1.51 (1.23, 1.87)		3.08 (2.54, 3.73)
Model 2		Reference		1.14 (0.91, 1.42)		1.77 (1.44, 2.17)
Model 3		Reference		1.17 (0.93, 1.46)		1.79 (1.44, 2.22)
Model 4		Reference		1.11 (0.77, 1.62)		1.33 (0.87, 2.06)
AS	275 (19.2)		629 (25.5)		1,274 (37.9)	
Model 1		Reference		1.44 (1.22, 1.68)		2.56 (2.21, 2.97)
Model 2		Reference		1.05 (0.88, 1.25)		1.38 (1.17, 1.63)
Model 3		Reference		1.06 (0.89, 1.27)		1.37 (1.15, 1.63)
Model 4		Reference		1.14 (0.85, 1.53)		1.36 (0.96, 1.92)

TIMT, thickened intima-media thickness; AS, atherosclerosis; CI, confidence interval; OR, odds ratio; SDR, the ratio of systolic and diastolic blood pressure.

Model 1 was crude; Model 2 was adjusted for age abd sex; Model 3 was adjusted for age, sex, BMI, occupation, education, alcohol drinking, smoking, diabetes, and dyslipidemia. Model 4 was adjusted for age, sex, BMI, occupation, education, alcohol drinking, smoking, diabetes, dyslipidemia, SBP, and treatment of AS.

### 3.5 Stratification analysis

In fully adjusted logistic models, compared to L-SDR, H-SDR was associated with increased risks of C-TIMT, C-AS, CF-TIMT, and CF-AS in the non-hypertensive population (Supplementary Table S2), while only C-TIMT, C-AS in the HT stratum (Supplementary Table S3). In the hypertensive population, the RCS between SDR and TIMT, plaque, or AS showed a significant linear trend, and *P* values for non-linear trend were 0.558, 0.168 and 0.361 (Supplementary Figures S1D–S1F). The risks of CF-TIMT, CF-P, and CF-AS increased with the rise of the SDR level. In the non-hypertensive population, RCS between SDR and CF-AS also showed the same results (Supplementary Figure S1I), but not for TIMT and plaque (Supplementary Figures S1G,H).

### 3.6 Sensitivity analysis

To exclude the potential effect of medication, we reanalyzed the association of SDR-AS among participants without taking any antihypertensive medicine. The increased risk of C-TIMT, C-AS, CF-TIMT, and CF-AS could also be observed in high-SDR when compared with low-SDR in multivariable-adjusted logistic models (Supplementary Table S4). The RCS between SDR and CF-AS also showed a significant linear trend, with SDR = 1.56 when OR = 1.0 (Supplementary Figure S2). In BP subgroups, both HT and PreHT were not associated with any risk of AS when compared with normotension (Supplementary Table S5). We also regrouped the population into six subgroups according to SDR tertiles and HT status, and their associations with risk for AS in logistic models are shown in Supplementary Table S6. H-SDR and non-HT were associated with increased risks of C-TIMT, C-AS, CF-TIMT, and CF-AS. H-SDR and HT were associated with higher C-AS, CF-TIMT, and CF-AS risks. On the contrary, this association was only significant among the non-hypertensive population (Supplementary Table S7), not among the hypertensive population (Supplementary Table S8). The RCSs in non-hypertensive and hypertensive populations were both linear, with SDR at 1.54 and 1.62 when OR = 1.0 (Supplementary Figure S3).

## 4 Discussion

The present study reported an AS screening project among native residents from a prospective cohort study in southeastern China and comprehensively investigated the association between BP and peripheral AS, including carotid and femoral arteries. The prevalence of AS, including TIMT and plaque, was 22.7% in the carotid artery, and 19.5% in the femoral artery. Of note, the total prevalence of AS was as high as 30.0% in either the carotid or femoral artery. The high SDR led to increased risks of TIMT, plaque, and AS, especially in the carotid artery. These findings suggested that SDR could provide additional predictive value for AS, regardless of hypertension or not. As a readily obtainable index, the clinical implication of SDR for AS or AS-related diseases is calling for further studies.

In the process of AS formation, the lesion from the fatty streak develops in the intima of the artery wall, thickening intima, which is an early sign of AS. Then the fatty streak evolves into a fibrous plaque. Thus, both TIMT and plaque were included in AS definition (17, 18). The sites of vessel branching in large-sized arteries are most vulnerable to AS (4). To date, the carotid artery is most frequently reported in previous studies (3), while the femoral artery has been less studied in a general population setting. In such cases, a screening program was launched among rural residents aged 35–74 years in southeastern China, and the IMT and presence of plaques were measured at the far wall of the left and right carotid arteries and femoral arteries concurrently (11, 12). Carotid AS, defined as TIMT and plaque, was reported among 22.7% of the current population, which was similar to the results of a meta-analysis using global datasets (3). As for femoral AS, it was diagnosed among 19.5% of the whole population, suggesting the AS screen in femoral arteries cannot be neglected. Previous studies, mostly from hospitalized populations, also showed the importance of additional femoral AS in cardiovascular risk assessment (19–21), and a study among factory workers in Spain found AS in 72% of the study population, with the prevalence of plaques in the femoral artery (54%), higher than that in the carotid artery (34%) (19). However, the importance of femoral AS in the general population is always underestimated. We highlighted more carotid and femoral AS screening programs in more generalized, larger sample-sized populations to address the burden of AS at finer levels.

HT could affect the arterial system by thickening artery walls, involving atherosclerotic plaques, and even increasing the vulnerability to rupture. Also, it has been widely recognized as an important, modifiable risk factor in CVD prevention (22). Previous observational studies have reported the significant positive association of HT with TIMT, plaque, and AS in carotid arteries (3, 23–25). Then we uniquely assessed these in the femoral artery, which was underdiagnosed. HT was positively associated with femoral plaque as previously reported (19, 26). Furthermore, a population-based cohort also reported the effect of elevated BP on hospitalized peripheral artery disease risk (27). Results from our study in the Chinese population provided supportive evidence regarding the role of HT on AS. In addition, we found that the risks of carotid or femoral TIMT, plaque, and AS were comparable between normotension and PreHT. Then a heatmap of ORs for AS risk in different SBP and DBP levels suggests there might be a complex interaction between SBP and DBP on AS prevalence. Thus, we inferred that an index combining SBP and DBP may provide more information on the role of blood pressure on AS.

SBP is mainly determined by cardiac output and proximal arterial capacity, while DBP is more likely to be affected by peripheral vascular volume and resistance (28, 29). Major arteries can store part of the stroke volume during systolic ejection, and drain this volume during diastole, which could ensure continuous perfusion of organs and tissues (30). A study on ambulatory BP monitoring (ABPM) assessment showed that SDR had a similar number to the “gold ratio" (31) with a value of 1.62 (32). In the present study, the mean of SDR was 1.59 ± 0.17, also around the “gold ratio”. The SDR was significantly, positively correlated to SBP with a coefficient of 0.515, and negatively to DBP but with a much lower coefficient (−0.197). Then we found that high tertile of SDR was significantly, and positively associated with TIMT, plaque, and AS risk in the carotid artery and carotid and/or femoral artery, but only AS in the femoral artery. In RCS analysis, a significant linear trend between SDR and HT was observed, and an SDR < 1.57 shared a relatively lower risk than those higher SDR. Given the effect of HT on AS, we regrouped all participants according to the tertiles of SDR and HT or not. It is noteworthy that increased risks for TIMT, plaque, and AS in the carotid artery and carotid and/or femoral artery were also observed in the high-SDR group in the absence of HT, indicating high-SDR may be a risk factor for AS independent of HT.

### 4.1 Limitations

SDR is a readily obtainable index, and our study first provided epidemiological evidence regarding the association between SDR and AS in carotid and femoral arteries. But the results should be interpreted with caution since the limitations were inevitable. First, although multiple statistical analyses and sensitivity analyses were used to confirm the association between SDR and AS, our cross-sectional design and sample size still limited the temporal interpretation or casual interference, thus further validation in populations with larger sample-sized longitudinal cohort studies is needed. Second, considering the variation of BP in different situations, BP readings in two or more days were recommended in HT diagnosis and BP evaluation in 2017 ACC/AHA guidelines (33). In the current study, two BP measurements in a single visit were conducted, which may lead to some false diagnoses of HT and BP evaluation. Third, the misclassification of TIMT, plaque, and AS could not be avoided due to the ultrasound subjective judgment of examiners. However, a total of five vascular surgeons with 3–5 years of clinical experience from the same department were trained to conduct all measurements, which we believe could increase the accuracy of AS measurement and reduce the probability of biased association as much as possible. However, residual bias from some other factors cannot be totally excluded, such as diet, physical activity, and so on.

## 5 Conclusions

AS is prevalent in middle-aged adults, with a prevalence of 30.0% in our current study population in southeastern China, and femoral AS consists of an important part of AS burden. SDR is linearly associated with the risk of AS, which is stronger in the carotid artery than in the femoral artery. These findings suggest that SDR can provide additional predictive value for AS, independent of hypertension.

## Data Availability

The original contributions presented in the study are included in the article/Supplementary Material, further inquiries can be directed to the corresponding authors.
