# Supplementary material for: The ratio of systolic and diastolic pressure is associated with carotid and femoral atherosclerosis
Source: Front Cardiovasc Med. 2024 Mar 8;11:1353945. doi: 10.3389/fcvm.2024.1353945 (PMC10957569; doi:10.3389/fcvm.2024.1353945)
Supplement: Supplementary file 1 [file Datasheet1.docx]

**SUPPLEMENTAL MATERIALS**

**Supplemental Table 1. The association of SDR-HT with AS risk.**

| **Variables** | **L-SDR & non-HT (n=1523)** | **M-SDR & non-HT (n=1489)** | **H-SDR & non-HT (n=887)** | **L-SDR & HT (n=899)** | **M-SDR & HT (n=928)** | **H-SDR & HT (n=1537)** | |  |  |
| --- | --- | --- | --- | --- | --- | --- | --- | --- | --- |
| **Carotid artery** | | | | | | | | |  |
| **TIMT** |  |  |  |  |  |  | |  |  |
| Model 1 | Reference | 1.37 (1.09,1.71) | 2.00 (1.58,2.54) | 1.86 (1.47,2.37) | 2.23 (1.77,2.82) | 3.27 (2.67,4.00) | |  |  |
| Model 2 | Reference | 1.22 (0.96,1.55) | 1.55 (1.20,2.00) | 1.34 (1.04,1.72) | 1.38 (1.07,1.76) | 1.81 (1.45,2.26) | |  |  |
| Model 3 | Reference | 1.23 (0.97,1.56) | 1.60 (1.23,2.06) | 1.30 (1.00,1.68) | 1.35 (1.05,1.74) | 1.74 (1.38,2.18) | |  |  |
| Model 4 | Reference | 1.18 (0.92,1.53) | 1.53 (1.16,2.03) | 1.25 (0.90,1.73) | 1.30 (0.91,1.85) | 1.61 (1.13,2.29) | |  |  |
| **Plaque** |  |  |  |  |  |  | |  |  |
| Model 1 | Reference | 1.10 (0.83,1.45) | 1.87 (1.41,2.49) | 2.04 (1.55,2.70) | 2.51 (1.92,3.27) | 3.60 (2.85,4.55) | |  |  |
| Model 2 | Reference | 0.96 (0.73,1.28) | 1.43 (1.06,1.92) | 1.50 (1.13,2.00) | 1.59 (1.20,2.11) | 2.03 (1.58,2.62) | |  |  |
| Model 3 | Reference | 0.98 (0.73,1.30) | 1.46 (1.08,1.97) | 1.51 (1.13,2.02) | 1.63 (1.23,2.17) | 2.00 (1.54,2.59) | |  |  |
| Model 4 | Reference | 0.91 (0.67,1.23) | 1.29 (0.93,1.79) | 1.10 (0.76,1.60) | 1.16 (0.78,1.72) | 1.40 (0.94,2.08) | |  |  |
| **AS** |  |  |  |  |  |  | |  |  |
| Model 1 | Reference | 1.43 (1.17,1.76) | 2.16 (1.74,2.69) | 2.15 (1.73,2.67) | 2.65 (2.15,3.27) | 3.87 (3.22,4.66) | |  |  |
| Model 2 | Reference | 1.28 (1.03,1.59) | 1.66 (1.31,2.11) | 1.56 (1.24,1.97) | 1.63 (1.30,2.05) | 2.10 (1.70,2.58) | |  |  |
| Model 3 | Reference | 1.29 (1.04,1.61) | 1.72 (1.35,2.18) | 1.54 (1.21,1.95) | 1.63 (1.29,2.05) | 2.03 (1.64,2.51) | |  |  |
| Model 4 | Reference | 1.25 (0.99,1.58) | 1.63 (1.26,2.12) | 1.35 (1.00,1.82) | 1.41 (1.02,1.95) | 1.73 (1.25,2.40) | |  |  |
| **Femoral artery** | | | | | | | | |  |
| **TIMT** |  |  |  |  |  |  | |  |  |
| Model 1 | Reference | 1.08 (0.87,1.35) | 1.34 (1.05,1.71) | 1.39 (1.09,1.77) | 1.54 (1.22,1.95) | 2.13 (1.74,2.60) | |  |  |
| Model 2 | Reference | 0.95 (0.75,1.20) | 1.01 (0.78,1.31) | 0.99 (0.77,1.27) | 0.94 (0.74,1.21) | 1.17 (0.94,1.47) | |  |  |
| Model 3 | Reference | 0.96 (0.76,1.21) | 1.04 (0.80,1.36) | 1.04 (0.80,1.34) | 1.00 (0.78,1.29) | 1.20 (0.96,1.51) | |  |  |
| Model 4 | Reference | 1.15 (0.90,1.48) | 1.33 (0.99,1.77) | 1.42 (1.02,1.99) | 1.43 (0.99,2.07) | 1.61 (1.12,2.33) | |  |  |
| **Plaque** |  |  |  |  |  |  | |  |  |
| Model 1 | Reference | 1.20 (0.90,1.59) | 1.29 (0.94,1.78) | 2.09 (1.57,2.79) | 2.44 (1.85,3.22) | 2.96 (2.31,3.78) | |  |  |
| Model 2 | Reference | 1.07 (0.80,1.44) | 0.99 (0.71,1.38) | 1.54 (1.15,2.07) | 1.58 (1.18,2.11) | 1.68 (1.28,2.19) | |  |  |
| Model 3 | Reference | 1.11 (0.83,1.49) | 1.05 (0.75,1.47) | 1.57 (1.16,2.13) | 1.68 (1.25,2.27) | 1.71 (1.30,2.25) | |  |  |
| Model 4 | Reference | 1.14 (0.83,1.55) | 1.09 (0.75,1.56) | 1.47 (0.99,2.18) | 1.44 (0.94,2.22) | 1.34 (0.87,2.07) | |  |  |
| **AS** |  |  |  |  |  |  | |  |  |
| Model 1 | Reference | 1.11 (0.90,1.36) | 1.41 (1.13,1.76) | 1.56 (1.25,1.94) | 1.92 (1.56,2.37) | 2.38 (1.98,2.85) | |  |  |
| Model 2 | Reference | 0.98 (0.79,1.21) | 1.07 (0.84,1.36) | 1.11 (0.88,1.41) | 1.20 (0.96,1.51) | 1.31 (1.06,1.61) | |  |  |
| Model 3 | Reference | 0.99 (0.80,1.23) | 1.11 (0.87,1.42) | 1.16 (0.91,1.47) | 1.28 (1.01,1.61) | 1.33 (1.08,1.65) | |  |  |
| Model 4 | Reference | 1.13 (0.89,1.42) | 1.31 (1.00,1.71) | 1.38 (1.02,1.87) | 1.52 (1.09,2.12) | 1.47 (1.05,2.06) | |  |  |
| **Carotid artery or Femoral artery** | | | | | | | | |  |
| **TIMT** |  |  |  |  |  |  | | | |
| Model 1 | Reference | 1.20 (0.99,1.44) | 1.64 (1.34,2.02) | 1.59 (1.29,1.95) | 1.86 (1.53,2.28) | 2.73 (2.30,3.24) |  |  |  |
| Model 2 | Reference | 1.05 (0.86,1.28) | 1.23 (0.98,1.54) | 1.11 (0.89,1.39) | 1.10 (0.88,1.37) | 1.42 (1.17,1.73) |  |  |  |
| Model 3 | Reference | 1.05 (0.86,1.29) | 1.26 (1.01,1.59) | 1.12 (0.89,1.41) | 1.11 (0.89,1.39) | 1.41 (1.15,1.72) |  |  |  |
| Model 4 | Reference | 1.17 (0.94,1.46) | 1.45 (1.14,1.86) | 1.39 (1.04,1.85) | 1.41 (1.03,1.93) | 1.70 (1.24,2.33) |  |  |  |
| **Plaque** |  |  |  |  |  |  |  |  |  |
| Model 1 | Reference | 1.19 (0.95,1.49) | 1.66 (1.30,2.12) | 2.09 (1.65,2.64) | 2.58 (2.06,3.23) | 3.49 (2.86,4.26) |  |  |  |
| Model 2 | Reference | 1.06 (0.83,1.34) | 1.27 (0.98,1.65) | 1.54 (1.20,1.97) | 1.66 (1.30,2.11) | 1.98 (1.59,2.47) |  |  |  |
| Model 3 | Reference | 1.08 (0.85,1.37) | 1.32 (1.02,1.72) | 1.55 (1.20,1.99) | 1.72 (1.35,2.20) | 1.97 (1.57,2.47) |  |  |  |
| Model 4 | Reference | 1.06 (0.82,1.37) | 1.27 (0.95,1.69) | 1.30 (0.94,1.80) | 1.34 (0.95,1.90) | 1.45 (1.02,2.06) |  |  |  |
| **AS** |  |  |  |  |  |  |  |  |  |
| Model 1 | Reference | 1.26 (1.06,1.50) | 1.81 (1.49,2.19) | 1.88 (1.55,2.27) | 2.32 (1.93,2.79) | 3.21 (2.73,3.78) |  |  |  |
| Model 2 | Reference | 1.12 (0.92,1.35) | 1.38 (1.12,1.71) | 1.35 (1.10,1.67) | 1.42 (1.16,1.75) | 1.71 (1.41,2.06) |  |  |  |
| Model 3 | Reference | 1.13 (0.93,1.37) | 1.43 (1.15,1.77) | 1.36 (1.10,1.68) | 1.44 (1.17,1.77) | 1.68 (1.38,2.03) |  |  |  |
| Model 4 | Reference | 1.20 (0.98,1.48) | 1.54 (1.21,1.95) | 1.46 (1.11,1.91) | 1.54 (1.15,2.08) | 1.72 (1.27,2.32) |  |  |  |

Note: TIMT, thickened intima-media thickness; AS, atherosclerosis; HT, hypertension; non-HT, none hypertension; L-SDR, low-SDR; M-SDR, medium-SDR; H-SDR, high-SDR; CI, confidence interval; OR, odds ratio; SDR, the ratio of systolic and diastolic blood pressure. Model 1 was crude; Model 2 was adjusted for age, sex; Model 3 was adjusted for age, sex, BMI, occupation, education, alcohol drinking, smoking, diabetes, and dyslipidemia; Model 4 was adjusted for age, sex, BMI, occupation, education, alcohol drinking, smoking, diabetes, dyslipidemia, SBP, and treatment of AS.

**Supplemental Table 2. The association between SDR and AS risk among non-hypertensive participants.**

| **Variables** | **L-SDR & non-HT** | |  | **M-SDR & non-HT** | |  | **H-SDR & non-HT** | |
| --- | --- | --- | --- | --- | --- | --- | --- | --- |
|  | **N (%)** | **OR (95% CI)** |  | **N (%)** | **OR (95% CI)** |  | **N (%)** | **OR (95% CI)** |
| **Carotid artery** | | | | | | | | |
| **TIMT** | 152 (10.0) |  |  | 196 (13.2) |  |  | 161 (18.2) |  |
| Model 1 |  | Reference |  |  | 1.37 (1.09,1.71) |  |  | 2.00 (1.58,2.54) |
| Model 2 |  | Reference |  |  | 1.22 (0.96,1.55) |  |  | 1.54 (1.19,2.00) |
| Model 3 |  | Reference |  |  | 1.23 (0.97,1.57) |  |  | 1.59 (1.22,2.06) |
| Model 4 |  | Reference |  |  | 1.16 (0.90,1.49) |  |  | 1.46 (1.10,1.94) |
| **Plaque** | 104 (6.8) |  |  | 111 (7.5) |  |  | 107 (12.1) |  |
| Model 1 |  | Reference |  |  | 1.10 (0.83,1.45) |  |  | 1.87 (1.41,2.49) |
| Model 2 |  | Reference |  |  | 0.96 (0.72,1.28) |  |  | 1.43 (1.06,1.93) |
| Model 3 |  | Reference |  |  | 0.98 (0.73,1.31) |  |  | 1.47 (1.09,1.99) |
| Model 4 |  | Reference |  |  | 0.92 (0.68,1.26) |  |  | 1.33 (0.95,1.86) |
| **AS** | 188 (12.3) |  |  | 250 (16.8) |  |  | 207 (23.3) |  |
| Model 1 |  | Reference |  |  | 1.43 (1.17,1.76) |  |  | 2.16 (1.74,2.69) |
| Model 2 |  | Reference |  |  | 1.27 (1.02,1.58) |  |  | 1.64 (1.29,2.08) |
| Model 3 |  | Reference |  |  | 1.29 (1.03,1.62) |  |  | 1.69 (1.32,2.16) |
| Model 4 |  | Reference |  |  | 1.23 (0.97,1.56) |  |  | 1.56 (1.19,2.03) |
| **Femoral artery** | | | | | | | | |
| **TIMT** | 173 (11.4) |  |  | 181 (12.2) |  |  | 130 (14.7) |  |
| Model 1 |  | Reference |  |  | 1.08 (0.87,1.35) |  |  | 1.34 (1.05,1.71) |
| Model 2 |  | Reference |  |  | 0.96 (0.76,1.21) |  |  | 1.03 (0.79,1.34) |
| Model 3 |  | Reference |  |  | 0.96 (0.76,1.22) |  |  | 1.06 (0.81,1.37) |
| Model 4 |  | Reference |  |  | 1.13 (0.88,1.46) |  |  | 1.28 (0.96,1.71) |
| **Plaque** | 96 (6.3) |  |  | 111 (7.5) |  |  | 71 (8.0) |  |
| Model 1 |  | Reference |  |  | 1.20 (0.90,1.59) |  |  | 1.29 (0.94,1.78) |
| Model 2 |  | Reference |  |  | 1.09 (0.81,1.47) |  |  | 1.02 (0.73,1.44) |
| Model 3 |  | Reference |  |  | 1.13 (0.84,1.53) |  |  | 1.10 (0.78,1.55) |
| Model 4 |  | Reference |  |  | 1.11 (0.81,1.52) |  |  | 1.05 (0.73,1.53) |
| **AS** | 210 (13.8) |  |  | 224 (15.0) |  |  | 163 (18.4) |  |
| Model 1 |  | Reference |  |  | 1.11 (0.90,1.36) |  |  | 1.41 (1.13,1.76) |
| Model 2 |  | Reference |  |  | 0.99 (0.79,1.23) |  |  | 1.09 (0.86,1.40) |
| Model 3 |  | Reference |  |  | 1.00 (0.80,1.25) |  |  | 1.13 (0.89,1.45) |
| Model 4 |  | Reference |  |  | 1.10 (0.87,1.39) |  |  | 1.26 (0.96,1.65) |
| **Carotid artery or Femoral artery** | | | | | | | | |
| **TIMT** | 247 (16.2) |  |  | 280 (18.8) |  |  | 214 (24.1) |  |
| Model 1 |  | Reference |  |  | 1.20 (0.99,1.44) |  |  | 1.64 (1.34,2.02) |
| Model 2 |  | Reference |  |  | 1.06 (0.86,1.30) |  |  | 1.25 (1.00,1.57) |
| Model 3 |  | Reference |  |  | 1.06 (0.86,1.30) |  |  | 1.28 (1.02,1.61) |
| Model 4 |  | Reference |  |  | 1.15 (0.92,1.43) |  |  | 1.39 (1.08,1.79) |
| **Plaque** | 156 (10.2) |  |  | 178 (12.0) |  |  | 141 (15.9) |  |
| Model 1 |  | Reference |  |  | 1.19 (0.95,1.50) |  |  | 1.66 (1.30,2.12) |
| Model 2 |  | Reference |  |  | 1.06 (0.83,1.35) |  |  | 1.28 (0.99,1.68) |
| Model 3 |  | Reference |  |  | 1.09 (0.85,1.39) |  |  | 1.36 (1.04,1.77) |
| Model 4 |  | Reference |  |  | 1.05 (0.81,1.36) |  |  | 1.27 (0.95,1.70) |
| **AS** | 293 (19.2) |  |  | 344 (23.1) |  |  | 267 (30.1) |  |
| Model 1 |  | Reference |  |  | 1.26 (1.06,1.50) |  |  | 1.81 (1.49,2.19) |
| Model 2 |  | Reference |  |  | 1.12 (0.92,1.36) |  |  | 1.38 (1.11,1.72) |
| Model 3 |  | Reference |  |  | 1.13 (0.93,1.38) |  |  | 1.43 (1.15,1.78) |
| Model 4 |  | Reference |  |  | 1.18 (0.96,1.46) |  |  | 1.48 (1.16,1.88) |

Note: TIMT: thickened intima-media thickness; AS: atherosclerosis; HT: hypertension; non-HT: none hypertension; L-SDR: low-SDR; M-SDR: medium-SDR; H-SDR: high-SDR; CI: confidence interval; OR: odds ratio; SDR: the ratio of systolic and diastolic blood pressure. Model 1 was crude; Model 2 was adjusted for age, sex; Model 3 was adjusted for age, sex, BMI, occupation, education, alcohol drinking, smoking, diabetes, and dyslipidemia; Model 4 was adjusted for age, sex, BMI, occupation, education, alcohol drinking, smoking, diabetes, dyslipidemia, SBP, and treatment of AS.

**Supplemental Table 3. The association between SDR and AS risk among hypertensive participants.**

| **Variables** | **L-SDR & HT** | |  | **M-SDR & HT** | |  | **H-SDR & HT** | |
| --- | --- | --- | --- | --- | --- | --- | --- | --- |
|  | **N (%)** | **OR (95% CI)** |  | **N (%)** | **OR (95% CI)** |  | **N (%)** | **OR (95% CI)** |
| **Carotid artery** | | | | | | | | |
| **TIMT** | 154 (17.1) |  |  | 184 (19.8) |  |  | 409 (26.6) |  |
| Model 1 |  | Reference |  |  | 1.20 (0.94,1.52) |  |  | 1.75 (1.43,2.16) |
| Model 2 |  | Reference |  |  | 1.02 (0.79,1.32) |  |  | 1.34 (1.06,1.69) |
| Model 3 |  | Reference |  |  | 1.03 (0.80,1.33) |  |  | 1.30 (1.02,1.65) |
| Model 4 |  | Reference |  |  | 1.05 (0.80,1.39) |  |  | 1.30 (1.00,1.73) |
| **Plaque** | 117 (13.0) |  |  | 144 (15.5) |  |  | 321 (20.9) |  |
| Model 1 |  | Reference |  |  | 1.23 (0.94,1.60) |  |  | 1.76 (1.40,2.22) |
| Model 2 |  | Reference |  |  | 1.06 (0.80,1.39) |  |  | 1.33 (1.03,1.71) |
| Model 3 |  | Reference |  |  | 1.08 (0.82,1.42) |  |  | 1.30 (1.00,1.68) |
| Model 4 |  | Reference |  |  | 1.00 (0.74,1.35) |  |  | 1.19 (0.89,1.61) |
| **AS** | 209 (23.3) |  |  | 252 (27.2) |  |  | 542 (35.3) |  |
| Model 1 |  | Reference |  |  | 1.23 (1.00,1.52) |  |  | 1.80 (1.49,2.17) |
| Model 2 |  | Reference |  |  | 1.05 (0.83,1.32) |  |  | 1.36 (1.10,1.69) |
| Model 3 |  | Reference |  |  | 1.06 (0.84,1.33) |  |  | 1.31 (1.05,1.63) |
| Model 4 |  | Reference |  |  | 1.07 (0.83,1.37) |  |  | 1.31 (1.02,1.69) |
| **Femoral artery** | | | | | | | | |
| **TIMT** | 136 (15.1) |  |  | 153 (16.5) |  |  | 329 (21.4) |  |
| Model 1 |  | Reference |  |  | 1.11 (0.86,1.43) |  |  | 1.53 (1.23,1.90) |
| Model 2 |  | Reference |  |  | 0.94 (0.72,1.22) |  |  | 1.14 (0.89,1.46) |
| Model 3 |  | Reference |  |  | 0.95 (0.72,1.24) |  |  | 1.10 (0.86,1.42) |
| Model 4 |  | Reference |  |  | 1.04 (0.77,1.40) |  |  | 1.17 (0.87,1.58) |
| **Plaque** | 111 (12.4) |  |  | 131 (14.1) |  |  | 255 (16.6) |  |
| Model 1 |  | Reference |  |  | 1.17 (0.89,1.53) |  |  | 1.41 (1.11,1.80) |
| Model 2 |  | Reference |  |  | 1.00 (0.75,1.33) |  |  | 1.03 (0.79,1.34) |
| Model 3 |  | Reference |  |  | 1.05 (0.79,1.41) |  |  | 1.03 (0.78,1.35) |
| Model 4 |  | Reference |  |  | 0.99 (0.72,1.36) |  |  | 0.91 (0.66,1.25) |
| **AS** | 179 (19.9) |  |  | 218 (23.5) |  |  | 423 (27.5) |  |
| Model 1 |  | Reference |  |  | 1.24 (0.99,1.54) |  |  | 1.53 (1.25,1.86) |
| Model 2 |  | Reference |  |  | 1.06 (0.83,1.34) |  |  | 1.12 (0.90,1.40) |
| Model 3 |  | Reference |  |  | 1.09 (0.85,1.39) |  |  | 1.09 (0.87,1.38) |
| Model 4 |  | Reference |  |  | 1.13 (0.86,1.47) |  |  | 1.08 (0.83,1.42) |
| **Carotid artery or Femoral artery** | | | | | | | | |
| **TIMT** | 211 (23.5) |  |  | 246 (26.5) |  |  | 531 (34.6) |  |
| Model 1 |  | Reference |  |  | 1.18 (0.95,1.45) |  |  | 1.72 (1.43,2.07) |
| Model 2 |  | Reference |  |  | 0.96 (0.76,1.21) |  |  | 1.22 (0.98,1.52) |
| Model 3 |  | Reference |  |  | 0.97 (0.76,1.22) |  |  | 1.18 (0.95,1.47) |
| Model 4 |  | Reference |  |  | 1.04 (0.81,1.34) |  |  | 1.25 (0.97,1.62) |
| **Plaque** | 173 (19.2) |  |  | 211 (22.7) |  |  | 438 (28.5) |  |
| Model 1 |  | Reference |  |  | 1.24 (0.99,1.55) |  |  | 1.67 (1.37,2.04) |
| Model 2 |  | Reference |  |  | 1.07 (0.84,1.35) |  |  | 1.25 (1.00,1.56) |
| Model 3 |  | Reference |  |  | 1.10 (0.86,1.41) |  |  | 1.23 (0.98,1.55) |
| Model 4 |  | Reference |  |  | 1.01 (0.78,1.32) |  |  | 1.08 (0.83,1.42) |
| **AS** | 278 (30.9) |  |  | 330 (35.6) |  |  | 666 (43.3) |  |
| Model 1 |  | Reference |  |  | 1.23 (1.01,1.50) |  |  | 1.71 (1.44,2.03) |
| Model 2 |  | Reference |  |  | 1.04 (0.84,1.29) |  |  | 1.24 (1.01,1.53) |
| Model 3 |  | Reference |  |  | 1.05 (0.84,1.30) |  |  | 1.20 (0.97,1.47) |
| Model 4 |  | Reference |  |  | 1.08 (0.85,1.37) |  |  | 1.20 (0.94,1.53) |

Note: TIMT, thickened intima-media thickness; AS, atherosclerosis; CI, confidence interval; OR, odds ratio; SDR, the ratio of systolic and diastolic blood pressure; L-SDR, low-SDR; M-SDR, medium-SDR; H-SDR, high-SDR. Model 1 was crude; Model 2 was adjusted for age, sex; Model 3 was adjusted for age, sex, BMI, occupation, education, alcohol drinking, smoking, diabetes, and dyslipidemia; Model 4 was adjusted for age, sex, BMI, occupation, education, alcohol drinking, smoking, diabetes, dyslipidemia, SBP, and treatment of AS.

**Supplemental Table 4. ORs and 95% CIs for the risk of AS by the Systolic/diastolic ratio of blood pressure in population without antihypertensive medication.**

| **Variables** | **Low SDR** | |  | **Medium SDR** | |  | **High SDR** | |
| --- | --- | --- | --- | --- | --- | --- | --- | --- |
|  | **N (%)** | **OR (95% CI)** |  | **N (%)** | **OR (95% CI)** |  | **N (%)** | **OR (95% CI)** |
| **Carotid artery** | | | | | | | | |
| **TIMT** | 227 (11.2) |  |  | 297 (14.5) |  |  | 431 (21.1) |  |
| Model 1 |  | Reference |  |  | 1.35 (1.12,1.62) |  |  | 2.13 (1.79,2.53) |
| Model 2 |  | Reference |  |  | 1.19 (0.98,1.45) |  |  | 1.48 (1.22,1.80) |
| Model 3 |  | Reference |  |  | 1.19 (0.98,1.45) |  |  | 1.49 (1.23,1.80) |
| Model 4 |  | Reference |  |  | 1.17 (0.95,1.43) |  |  | 1.43 (1.16,1.76) |
| **Plaque** | 167 (8.2) |  |  | 194 (9.5) |  |  | 299 (14.6) |  |
| Model 1 |  | Reference |  |  | 1.17 (0.94,1.45) |  |  | 1.92 (1.57,2.34) |
| Model 2 |  | Reference |  |  | 1.02 (0.81,1.28) |  |  | 1.34 (1.08,1.66) |
| Model 3 |  | Reference |  |  | 1.03 (0.82,1.29) |  |  | 1.35 (1.08,1.67) |
| Model 4 |  | Reference |  |  | 0.94 (0.74,1.18) |  |  | 1.12 (0.88,1.42) |
| **AS** | 297 (14.6) |  |  | 389 (19.0) |  |  | 557 (27.2) |  |
| Model 1 |  | Reference |  |  | 1.37 (1.16,1.62) |  |  | 2.19 (1.87,2.56) |
| Model 2 |  | Reference |  |  | 1.20 (1.01,1.44) |  |  | 1.50 (1.26,1.79) |
| Model 3 |  | Reference |  |  | 1.21 (1.01,1.45) |  |  | 1.50 (1.26,1.79) |
| Model 4 |  | Reference |  |  | 1.14 (0.95,1.38) |  |  | 1.35 (1.12,1.64) |
| **Femoral artery** | | | | | | | | |
| **TIMT** | 237 (11.6) |  |  | 271 (13.2) |  |  | 338 (16.5) |  |
| Model 1 |  | Reference |  |  | 1.15 (0.96,1.39) |  |  | 1.50 (1.26,1.80) |
| Model 2 |  | Reference |  |  | 1.02 (0.83,1.24) |  |  | 1.05 (0.86,1.27) |
| Model 3 |  | Reference |  |  | 1.02 (0.84,1.24) |  |  | 1.06 (0.87,1.29) |
| Model 4 |  | Reference |  |  | 1.10 (0.90,1.35) |  |  | 1.16 (0.93,1.44) |
| **Plaque** | 156 (7.7) |  |  | 181 (8.8) |  |  | 216 (10.6) |  |
| Model 1 |  | Reference |  |  | 1.17 (0.93,1.46) |  |  | 1.42 (1.15,1.76) |
| Model 2 |  | Reference |  |  | 1.04 (0.83,1.32) |  |  | 1.02 (0.80,1.28) |
| Model 3 |  | Reference |  |  | 1.07 (0.84,1.30) |  |  | 1.06 (0.84,1.34) |
| Model 4 |  | Reference |  |  | 1.00 (0.78,1.27) |  |  | 0.90 (0.69,1.16) |
| **AS** | 300 (14.7) |  |  | 344 (16.8) |  |  | 425 (20.8) |  |
| Model 1 |  | Reference |  |  | 1.17 (0.98,1.38) |  |  | 1.52 (1.29,1.78) |
| Model 2 |  | Reference |  |  | 1.03 (0.86,1.24) |  |  | 1.07 (0.89,1.28) |
| Model 3 |  | Reference |  |  | 1.04 (0.87,1.25) |  |  | 1.09 (0.91,1.31) |
| Model 4 |  | Reference |  |  | 1.07 (0.89,1.29) |  |  | 1.10 (0.90,1.34) |
| **Carotid artery or Femoral artery** | | | | | | | | |
| **TIMT** | 355 (17.4) |  |  | 418 (20.4) |  |  | 566 (27.7) |  |
| Model 1 |  | Reference |  |  | 1.21 (1.04,1.42) |  |  | 1.81 (1.56,2.10) |
| Model 2 |  | Reference |  |  | 1.05 (0.89,1.25) |  |  | 1.22 (1.03,1.45) |
| Model 3 |  | Reference |  |  | 1.05 (0.89,1.25) |  |  | 1.23 (1.04,1.46) |
| Model 4 |  | Reference |  |  | 1.09 (0.92,1.31) |  |  | 1.27 (1.05,1.53) |
| **Plaque** | 255 (12.5) |  |  | 297 (14.5) |  |  | 406 (19.8) |  |
| Model 1 |  | Reference |  |  | 1.18 (0.99,1.41) |  |  | 1.73 (1.46,2.05) |
| Model 2 |  | Reference |  |  | 1.04 (0.86,1.26) |  |  | 1.22 (1.01,1.47) |
| Model 3 |  | Reference |  |  | 1.06 (0.87,1.29) |  |  | 1.25 (1.03,1.51) |
| Model 4 |  | Reference |  |  | 0.96 (0.79,1.18) |  |  | 1.02 (0.83,1.25) |
| **AS** | 444 (21.8) |  |  | 527 (25.7) |  |  | 704 (34.4) |  |
| Model 1 |  | Reference |  |  | 1.24 (1.07,1.43) |  |  | 1.88 (1.64,2.16) |
| Model 2 |  | Reference |  |  | 1.09 (0.93,1.27) |  |  | 1.29 (1.09,1.51) |
| Model 3 |  | Reference |  |  | 1.09 (0.93,1.28) |  |  | 1.29 (1.10,1.52) |
| Model 4 |  | Reference |  |  | 1.07 (0.91,1.27) |  |  | 1.22 (1.03,1.46) |

Note: TIMT, thickened intima-media thickness; AS, atherosclerosis; CI, confidence interval; OR, odds ratio; SDR, the ratio of systolic and diastolic blood pressure. Logistic regression model was fully adjusted for age, sex, BMI, occupation, education, alcohol drinking, smoking, diabetes, dyslipidemia, SBP, and treatment of AS.

**Supplemental Table 5. The association between BP subgroups and AS risk in population without antihypertensive medication.**

| **Variables** | **Normotension** | |  | **Prehypertension** | |  | **Hypertension** | |
| --- | --- | --- | --- | --- | --- | --- | --- | --- |
|  | **N (%)** | **OR (95% CI)** |  | **N (%)** | **OR (95% CI)** |  | **N (%)** | **OR (95% CI)** |
| **Carotid artery** | | | | | | | | |
| **TIMT** | 139 (9.7) |  |  | 370 (15.0) |  |  | 446 (19.9) |  |
| Model 1 |  | Reference |  |  | 1.64 (1.33,2.01) |  |  | 2.31 (1.89,2.84) |
| Model 2 |  | Reference |  |  | 1.23 (0.99,1.52) |  |  | 1.33 (1.07,1.65) |
| Model 3 |  | Reference |  |  | 1.23 (0.98,1.53) |  |  | 1.30 (1.04,1.63) |
| Model 4 |  | Reference |  |  | 0.94 (0.64,1.36) |  |  | 0.88 (0.55,1.39) |
| **Plaque** | 87 (6.1) |  |  | 235 (9.5) |  |  | 338 (15.1) |  |
| Model 1 |  | Reference |  |  | 1.62 (1.26,2.10) |  |  | 2.75 (2.15,3.51) |
| Model 2 |  | Reference |  |  | 1.23 (0.95,1.60) |  |  | 1.67 (1.29,2.15) |
| Model 3 |  | Reference |  |  | 1.25 (0.96,1.63) |  |  | 1.68 (1.29,2.19) |
| Model 4 |  | Reference |  |  | 1.07 (0.67,1.70) |  |  | 0.98 (0.56,1.73) |
| **AS** | 174 (12.2) |  |  | 471 (19.1) |  |  | 598 (26.7) |  |
| Model 1 |  | Reference |  |  | 1.70 (1.41,2.05) |  |  | 2.63 (2.19,3.17) |
| Model 2 |  | Reference |  |  | 1.27 (1.04,1.55) |  |  | 1.52 (1.24,1.85) |
| Model 3 |  | Reference |  |  | 1.28 (1.05,1.57) |  |  | 1.50 (1.22,1.85) |
| Model 4 |  | Reference |  |  | 1.02 (0.72,1.46) |  |  | 0.95 (0.62,1.47) |
| **Femoral artery** | | | | | | | | |
| **TIMT** | 174 (12.2) |  |  | 310 (12.6) |  |  | 362 (16.2) |  |
| Model 1 |  | Reference |  |  | 1.04 (0.85,1.26) |  |  | 1.39 (1.15,1.69) |
| Model 2 |  | Reference |  |  | 0.76 (0.62,0.94) |  |  | 0.80 (0.65,0.98) |
| Model 3 |  | Reference |  |  | 0.78 (0.63,0.96) |  |  | 0.83 (0.67,1.03) |
| Model 4 |  | Reference |  |  | 1.02 (0.72,1.46) |  |  | 0.95 (0.62,1.47) |
| **Plaque** | 81 (5.7) |  |  | 197 (8.0) |  |  | 275 (12.3) |  |
| Model 1 |  | Reference |  |  | 1.44 (1.11,1.89) |  |  | 2.33 (1.81,3.02) |
| Model 2 |  | Reference |  |  | 1.09 (0.83,1.44) |  |  | 1.42 (1.08,1.85) |
| Model 3 |  | Reference |  |  | 1.14 (0.86,1.51) |  |  | 1.48 (1.12,1.95) |
| Model 4 |  | Reference |  |  | 1.15 (0.70,1.89) |  |  | 1.22 (0.67,2.24) |
| **AS** | 201 (14.1) |  |  | 396 (16.0) |  |  | 472 (21.1) |  |
| Model 1 |  | Reference |  |  | 1.17 (0.97,1.40) |  |  | 1.64 (1.37,1.96) |
| Model 2 |  | Reference |  |  | 0.86 (0.71,1.05) |  |  | 0.95 (0.78,1.15) |
| Model 3 |  | Reference |  |  | 0.88 (0.72,1.08) |  |  | 0.98 (0.80,1.20) |
| Model 4 |  | Reference |  |  | 0.99 (0.70,1.40) |  |  | 0.97 (0.62,1.50) |
| **Carotid artery or Femoral artery** | | | | | | | | |
| **TIMT** | 235 (16.4) |  |  | 506 (20.5) |  |  | 598 (26.7) |  |
| Model 1 |  | Reference |  |  | 1.31 (1.11,1.56) |  |  | 1.86 (1.57,2.20) |
| Model 2 |  | Reference |  |  | 0.95 (0.79,1.14) |  |  | 1.03 (0.85,1.24) |
| Model 3 |  | Reference |  |  | 0.96 (0.79,1.15) |  |  | 1.02 (0.84,1.24) |
| Model 4 |  | Reference |  |  | 1.02 (0.74,1.41) |  |  | 1.07 (0.71,1.61) |
| **Plaque** | 136 (9.5) |  |  | 339 (13.7) |  |  | 483 (21.6) |  |
| Model 1 |  | Reference |  |  | 1.51 (1.23,1.87) |  |  | 2.62 (2.14,3.21) |
| Model 2 |  | Reference |  |  | 1.14 (0.91,1.42) |  |  | 1.59 (1.28,1.97) |
| Model 3 |  | Reference |  |  | 1.17 (0.93,1.46) |  |  | 1.61 (1.29,2.02) |
| Model 4 |  | Reference |  |  | 1.01 (0.68,1.49) |  |  | 1.00 (0.62,1.62) |
| **AS** | 275 (19.2) |  |  | 629 (25.5) |  |  | 771 (34.5) |  |
| Model 1 |  | Reference |  |  | 1.44 (1.22,1.68) |  |  | 2.21 (1.89,2.59) |
| Model 2 |  | Reference |  |  | 1.05 (0.88,1.25) |  |  | 1.26 (1.05,1.50) |
| Model 3 |  | Reference |  |  | 1.06 (0.88,1.27) |  |  | 1.24 (1.04,1.49) |
| Model 4 |  | Reference |  |  | 1.03 (0.76,1.40) |  |  | 1.07 (0.73,1.58) |

Note: TIMT, thickened intima-media thickness; AS, atherosclerosis; CI, confidence interval; OR, odds ratio; SDR, the ratio of systolic and diastolic blood pressure. Model 1 was crude; Model 2 was adjusted for age, sex; Model 3 was adjusted for age, sex, BMI, occupation, education, drinking, smoking, diabetes, and dyslipidemia; Model 4 was adjusted for age, sex, BMI, occupation, education, alcohol drinking, smoking, diabetes, dyslipidemia, SBP, and treatment of AS.

**Supplemental Table 6. The association of SDR-HT and AS risk in population without antihypertensive medication.**

| **Variables** | **L-SDR & non-HT (N=1440)** | **M-SDR & non-HT (N=1477)** | **H-SDR & non-HT (N=982)** | **L-SDR & HT (N=596)** | **M-SDR & HT (N=576)** | **H-SDR & HT (N=1065)** |
| --- | --- | --- | --- | --- | --- | --- |
| **Carotid artery** | | | | | | |
| **TIMT** |  |  |  |  |  |  |
| Model 1 | Reference | 1.44 (1.14,1.81) | 2.06 (1.62,2.62) | 1.66 (1.25,2.20) | 2.03 (1.54,2.68) | 2.97 (2.37,3.72) |
| Model 2 | Reference | 1.27 (1.00,1.62) | 1.53 (1.19,1.98) | 1.23 (0.91,1.67) | 1.30 (0.97,1.75) | 1.65 (1.29,2.11) |
| Model 3 | Reference | 1.28 (1.00,1.63) | 1.57 (1.21,2.03) | 1.22 (0.90,1.65) | 1.29 (0.96,1.74) | 1.61 (1.26,2.07) |
| Model 4 | Reference | 1.21 (0.94,1.57) | 1.47 (1.11,1.95) | 1.10 (0.75,1.60) | 1.24 (0.80,1.91) | 1.49 (0.98,2.31) |
| **Plaque** |  |  |  |  |  |  |
| Model 1 | Reference | 1.10 (0.83,1.46) | 1.80 (1.36,2.39) | 1.79 (1.30,2.48) | 2.34 (1.72,3.19) | 2.88 (2.22,3.73) |
| Model 2 | Reference | 0.96 (0.72,1.29) | 1.33 (0.99,1.79) | 1.37 (0.98,1.91) | 1.56 (1.13,2.15) | 1.67 (1.26,2.20) |
| Model 3 | Reference | 0.97 (0.73,1.30) | 1.36 (1.01,1.84) | 1.38 (0.98,1.93) | 1.59 (1.15,2.20) | 1.67 (1.26,2.21) |
| Model 4 | Reference | 0.90 (0.66,1.23) | 1.18 (0.85,1.65) | 0.90 (0.58,1.40) | 0.99 (0.61,1.61) | 1.07 (0.66,1.73) |
| **Atherosclerosis** |  |  |  |  |  |  |
| Model 1 | Reference | 1.46 (1.19,1.80) | 2.15 (1.73,2.67) | 1.89 (1.47,2.44) | 2.38 (1.86,3.05) | 3.31 (2.70,4.06) |
| Model 2 | Reference | 1.29 (1.03,1.62) | 1.59 (1.25,2.01) | 1.43 (1.09,1.88) | 1.54 (1.18,2.01) | 1.81 (1.44,2.27) |
| Model 3 | Reference | 1.31 (1.04,1.64) | 1.62 (1.28,2.06) | 1.43 (1.09,1.88) | 1.55 (1.18,2.02) | 1.80 (1.43,2.27) |
| Model 4 | Reference | 1.24 (0.97,1.58) | 1.48 (1.14,1.93) | 1.13 (0.79,1.60) | 1.25 (0.84,1.86) | 1.47 (1.00,2.18) |
| **Femoral artery** | | | | | | |
| **TIMT** |  |  |  |  |  |  |
| Model 1 | Reference | 1.14 (0.91,1.43) | 1.39 (1.10,1.78) | 1.24 (0.93,1.66) | 1.48 (1.12,1.96) | 1.81 (1.44,2.27) |
| Model 2 | Reference | 1.00 (0.79,1.27) | 1.02 (0.79,1.32) | 0.92 (0.68,1.24) | 0.95 (0.71,1.28) | 1.01 (0.78,1.29) |
| Model 3 | Reference | 1.01 (0.80,1.28) | 1.05 (0.81,1.36) | 0.96 (0.71,1.30) | 0.99 (0.74,1.34) | 1.04 (0.80,1.33) |
| Model 4 | Reference | 1.21 (0.93,1.56) | 1.31 (0.99,1.75) | 1.19 (0.80,1.76) | 1.33 (0.84,2.09) | 1.26 (0.80,1.98) |
| **Plaque** |  |  |  |  |  |  |
| Model 1 | Reference | 1.20 (0.90,1.60) | 1.37 (1.00,1.87) | 1.92 (1.38,2.68) | 2.20 (1.59,3.05) | 2.20 (1.67,2.92) |
| Model 2 | Reference | 1.07 (0.79,1.45) | 1.03 (0.74,1.43) | 1.44 (1.02,2.04) | 1.48 (1.06,2.09) | 1.29 (0.96,1.75) |
| Model 3 | Reference | 1.10 (0.81,1.49) | 1.10 (0.79,1.52) | 1.47 (1.04,2.09) | 1.55 (1.09,2.19) | 1.35 (1.00,1.84) |
| Model 4 | Reference | 1.09 (0.79,1.51) | 1.07 (0.75,1.54) | 1.22 (0.77,1.94) | 1.13 (0.66,1.93) | 0.90 (0.52,1.53) |
| **Atherosclerosis** |  |  |  |  |  |  |
| Model 1 | Reference | 1.14 (0.92,1.40) | 1.45 (1.16,1.81) | 1.39 (1.07,1.80) | 1.73 (1.34,2.22) | 1.91 (1.55,2.35) |
| Model 2 | Reference | 1.00 (0.80,1.25) | 1.08 (0.85,1.37) | 1.03 (0.78,1.35) | 1.13 (0.86,1.48) | 1.08 (0.85,1.35) |
| Model 3 | Reference | 1.02 (0.81,1.27) | 1.12 (0.88,1.42) | 1.06 (0.80,1.40) | 1.18 (0.90,1.54) | 1.11 (0.88,1.41) |
| Model 4 | Reference | 1.13 (0.89,1.43) | 1.26 (0.97,1.65) | 1.14 (0.79,1.63) | 1.25 (0.82,1.89) | 1.09 (0.72,1.66) |
| **Carotid artery and Femoral artery** | | | | | | |
| **TIMT** |  |  |  |  |  |  |
| Model 1 | Reference | 1.24 (1.02,1.50) | 1.69 (1.38,2.07) | 1.46 (1.15,1.86) | 1.72 (1.35,2.18) | 2.40 (1.98,2.91) |
| Model 2 | Reference | 1.08 (0.88,1.33) | 1.22 (0.98,1.53) | 1.07 (0.82,1.39) | 1.07 (0.83,1.38) | 1.27 (1.02,1.58) |
| Model 3 | Reference | 1.09 (0.88,1.34) | 1.25 (1.00,1.57) | 1.09 (0.83,1.41) | 1.07 (0.83,1.39) | 1.26 (1.01,1.57) |
| Model 4 | Reference | 1.20 (0.96,1.50) | 1.41 (1.10,1.81) | 1.27 (0.91,1.78) | 1.34 (0.91,1.98) | 1.47 (1.00,2.16) |
| **Plaque** |  |  |  |  |  |  |
| Model 1 | Reference | 1.17 (0.92,1.47) | 1.65 (1.29,2.10) | 1.95 (1.49,2.55) | 2.41 (1.86,3.14) | 2.71 (2.17,3.39) |
| Model 2 | Reference | 1.03 (0.81,1.31) | 1.23 (0.95,1.60) | 1.48 (1.12,1.97) | 1.63 (1.24,2.16) | 1.58 (1.24,2.02) |
| Model 3 | Reference | 1.05 (0.82,1.34) | 1.29 (0.99,1.67) | 1.50 (1.12,2.00) | 1.67 (1.26,2.22) | 1.60 (1.25,2.05) |
| Model 4 | Reference | 1.01 (0.77,1.31) | 1.19 (0.89,1.59) | 1.11 (0.76,1.62) | 1.07 (0.70,1.64) | 0.99 (0.64,1.51) |
| **Atherosclerosis** |  |  |  |  |  |  |
| Model 1 | Reference | 1.28 (1.07,1.53) | 1.83 (1.51,2.21) | 1.74 (1.40,2.17) | 2.08 (1.67,2.59) | 2.70 (2.25,3.23) |
| Model 2 | Reference | 1.13 (0.93,1.37) | 1.35 (1.09,1.67) | 1.31 (1.03,1.67) | 1.34 (1.05,1.71) | 1.46 (1.19,1.80) |
| Model 3 | Reference | 1.14 (0.93,1.39) | 1.39 (1.12,1.72) | 1.31 (1.03,1.68) | 1.35 (1.06,1.72) | 1.45 (1.18,1.79) |
| Model 4 | Reference | 1.19 (0.96,1.47) | 1.44 (1.14,1.83) | 1.30 (0.95,1.79) | 1.32 (1.00,1.91) | 1.38 (1.01,1.98) |

Note: TIMT, thickened intima-media thickness; AS, atherosclerosis; CI, confidence interval; OR, odds ratio; SDR, the ratio of systolic and diastolic blood pressure. Model 1 was crude; Model 2 was adjusted for age, sex; Model 3 was adjusted for age, sex, BMI, occupation, education, alcohol drinking, smoking, diabetes, and dyslipidemia; Model 4 was adjusted for age, sex, BMI, occupation, education, alcohol drinking, smoking, diabetes, dyslipidemia, SBP, and treatment of AS.

**Supplemental Table 7. The association between SDR and AS risk among non-hypertensive participants in population without antihypertensive medication.**

| **Variables** | **L-SDR & non-HT** | |  | **M-SDR & non-HT** | |  | **H-SDR & non-HT** | |
| --- | --- | --- | --- | --- | --- | --- | --- | --- |
|  | **N (%)** | **OR (95% CI)** |  | **N (%)** | **OR (95% CI)** |  | **N (%)** | **OR (95% CI)** |
| **Carotid artery** | | | | | | | | |
| **TIMT** | 138 (9.6) |  |  | 195 (13.2) |  |  | 176 (17.9) |  |
| Model 1 |  | Reference |  |  | 1.44 (1.14,1.81) |  |  | 2.06 (1.62,2.62) |
| Model 2 |  | Reference |  |  | 1.28 (1.00,1.63) |  |  | 1.53 (1.18,1.99) |
| Model 3 |  | Reference |  |  | 1.28 (1.00,1.64) |  |  | 1.57 (1.21,2.04) |
| Model 4 |  | Reference |  |  | 1.21 (0.93,1.57) |  |  | 1.45 (1.09,1.92) |
| **Plaque** | 98 (6.8) |  |  | 110 (7.5) |  |  | 114 (11.6) |  |
| Model 1 |  | Reference |  |  | 1.10 (0.83,1.46) |  |  | 1.80 (1.36,2.39) |
| Model 2 |  | Reference |  |  | 0.95 (0.71,1.28) |  |  | 1.32 (0.98,1.78) |
| Model 3 |  | Reference |  |  | 0.97 (0.72,1.30) |  |  | 1.36 (1.00,1.84) |
| Model 4 |  | Reference |  |  | 0.91 (0.67,1.25) |  |  | 1.22 (0.87,1.70) |
| **AS** | 174 (12.1) |  |  | 247 (16.7) |  |  | 224 (22.8) |  |
| Model 1 |  | Reference |  |  | 1.46 (1.19,1.80) |  |  | 2.15 (1.73,2.67) |
| Model 2 |  | Reference |  |  | 1.29 (1.03,1.61) |  |  | 1.56 (1.23,1.98) |
| Model 3 |  | Reference |  |  | 1.30 (1.04,1.64) |  |  | 1.60 (1.26,2.04) |
| Model 4 |  | Reference |  |  | 1.24 (0.97,1.58) |  |  | 1.47 (1.13,1.91) |
| **Femoral artery** | | | | | | | | |
| **TIMT** | 158 (11.0) |  |  | 182 (12.3) |  |  | 144 (14.7) |  |
| Model 1 |  | Reference |  |  | 1.14 (0.91,1.43) |  |  | 1.39 (1.10,1.78) |
| Model 2 |  | Reference |  |  | 1.01 (0.79,1.28) |  |  | 1.04 (0.80,1.35) |
| Model 3 |  | Reference |  |  | 1.01 (0.80,1.29) |  |  | 1.07 (0.82,1.38) |
| Model 4 |  | Reference |  |  | 1.21 (0.93,1.56) |  |  | 1.31 (0.99,1.75) |
| **Plaque** | 89 (6.2) |  |  | 108 (7.3) |  |  | 81 (8.3) |  |
| Model 1 |  | Reference |  |  | 1.20 (0.90,1.60) |  |  | 1.37 (1.00,1.87) |
| Model 2 |  | Reference |  |  | 1.08 (0.80,1.46) |  |  | 1.04 (0.75,1.45) |
| Model 3 |  | Reference |  |  | 1.11 (0.82,1.51) |  |  | 1.11 (0.80,1.56) |
| Model 4 |  | Reference |  |  | 1.09 (0.79,1.51) |  |  | 1.07 (0.74,1.54) |
| **AS** | 194 (13.5) |  |  | 222 (15.0) |  |  | 181 (18.4) |  |
| Model 1 |  | Reference |  |  | 1.14 (0.92,1.40) |  |  | 1.45 (1.16,1.81) |
| Model 2 |  | Reference |  |  | 1.01 (0.81,1.26) |  |  | 1.09 (0.86,1.39) |
| Model 3 |  | Reference |  |  | 1.02 (0.82,1.28) |  |  | 1.13 (0.88,1.44) |
| Model 4 |  | Reference |  |  | 1.13 (0.89,1.44) |  |  | 1.27 (0.97,1.65) |
| **Carotid artery and Femoral artery** | | | | | | | | |
| **TIMT** | 227 (15.8) |  |  | 278 (18.8) |  |  | 236 (24.0) |  |
| Model 1 |  | Reference |  |  | 1.24 (1.02,1.50) |  |  | 1.69 (1.38,2.07) |
| Model 2 |  | Reference |  |  | 1.09 (0.89,1.35) |  |  | 1.25 (0.99,1.56) |
| Model 3 |  | Reference |  |  | 1.09 (0.89,1.35) |  |  | 1.27 (1.01,1.59) |
| Model 4 |  | Reference |  |  | 1.19 (0.95,1.49) |  |  | 1.39 (1.08,1.79) |
| **Plaque** | 147 (10.2) |  |  | 173 (11.7) |  |  | 155 (15.8) |  |
| Model 1 |  | Reference |  |  | 1.17 (0.93,1.47) |  |  | 1.65 (1.30,2.10) |
| Model 2 |  | Reference |  |  | 1.02 (0.80,1.31) |  |  | 1.23 (0.94,1.60) |
| Model 3 |  | Reference |  |  | 1.05 (0.81,1.34) |  |  | 1.29 (0.99,1.68) |
| Model 4 |  | Reference |  |  | 1.01 (0.78,1.32) |  |  | 1.20 (0.90,1.61) |
| **AS** | 272 (18.9) |  |  | 339 (23.0) |  |  | 293 (29.8) |  |
| Model 1 |  | Reference |  |  | 1.28 (1.07,1.53) |  |  | 1.83 (1.51,2.21) |
| Model 2 |  | Reference |  |  | 1.13 (0.92,1.37) |  |  | 1.35 (1.08,1.67) |
| Model 3 |  | Reference |  |  | 1.14 (0.93,1.39) |  |  | 1.38 (1.11,1.71) |
| Model 4 |  | Reference |  |  | 1.19 (0.96,1.47) |  |  | 1.43 (1.13,1.81) |

Note: TIMT, thickened intima-media thickness; AS, atherosclerosis; CI, confidence interval; OR, odds ratio; SDR, the ratio of systolic and diastolic blood pressure. Model 1 was crude; Model 2 was adjusted for age, sex; Model 3 was adjusted for age, sex, BMI, occupation, education, alcohol drinking, smoking, diabetes, and dyslipidemia; Model 4 was adjusted for age, sex, BMI, occupation, education, alcohol drinking, smoking, diabetes, dyslipidemia, SBP, and treatment of AS.

**Supplemental Table 8. The association between SDR and AS risk among hypertensive participants in population without antihypertensive medication.**

| **Variables** | **L-SDR & HT** | |  | **M-SDR & HT** | |  | **H-SDR & HT** | |
| --- | --- | --- | --- | --- | --- | --- | --- | --- |
|  | **N (%)** | **OR (95% CI)** |  | **N (%)** | **OR (95% CI)** |  | **N (%)** | **OR (95% CI)** |
| **Carotid artery** | | | | | | | | |
| **TIMT** | 89 (14.9) |  |  | 102 (17.7) |  |  | 255 (23.9) |  |
| Model 1 |  | Reference |  |  | 1.23 (0.90,1.67) |  |  | 1.79 (1.38,2.34) |
| Model 2 |  | Reference |  |  | 1.05 (0.76,1.46) |  |  | 1.31 (0.97,1.77) |
| Model 3 |  | Reference |  |  | 1.06 (0.76,1.48) |  |  | 1.27 (0.94,1.72) |
| Model 4 |  | Reference |  |  | 1.12 (0.77,1.63) |  |  | 1.34 (0.93,1.94) |
| **Plaque** | 69 (11.6) |  |  | 84 (14.6) |  |  | 185 (17.4) |  |
| Model 1 |  | Reference |  |  | 1.30 (0.93,1.83) |  |  | 1.61 (1.19,2.16) |
| Model 2 |  | Reference |  |  | 1.15 (0.80,1.64) |  |  | 1.22 (0.88,1.69) |
| Model 3 |  | Reference |  |  | 1.15 (0.81,1.65) |  |  | 1.20 (0.86,1.68) |
| Model 4 |  | Reference |  |  | 1.03 (0.69,1.53) |  |  | 1.10 (0.75,1.63) |
| **AS** | 123 (20.6) |  |  | 142 (24.7) |  |  | 333 (31.3) |  |
| Model 1 |  | Reference |  |  | 1.26 (0.96,1.66) |  |  | 1.75 (1.38,2.22) |
| Model 2 |  | Reference |  |  | 1.09 (0.81,1.46) |  |  | 1.30 (0.99,1.70) |
| Model 3 |  | Reference |  |  | 1.09 (0.81,1.47) |  |  | 1.25 (0.95,1.65) |
| Model 4 |  | Reference |  |  | 1.10 (0.79,1.53) |  |  | 1.28 (0.92,1.77) |
| **Femoral artery** | | | | | | | | |
| **TIMT** | 79 (13.3) |  |  | 89 (15.5) |  |  | 194 (18.2) |  |
| Model 1 |  | Reference |  |  | 1.20 (0.86,1.66) |  |  | 1.46 (1.10,1.94) |
| Model 2 |  | Reference |  |  | 1.02 (0.72,1.43) |  |  | 1.03 (0.75,1.41) |
| Model 3 |  | Reference |  |  | 1.03 (0.73,1.46) |  |  | 1.01 (0.73,1.39) |
| Model 4 |  | Reference |  |  | 1.13 (0.77,1.68) |  |  | 1.08 (0.73,1.60) |
| **Plaque** | 67 (11.2) |  |  | 73 (12.7) |  |  | 135 (12.7) |  |
| Model 1 |  | Reference |  |  | 1.15 (0.81,1.63) |  |  | 1.15 (0.84,1.57) |
| Model 2 |  | Reference |  |  | 1.02 (0.70,1.47) |  |  | 0.85 (0.60,1.21) |
| Model 3 |  | Reference |  |  | 1.06 (0.72,1.54) |  |  | 0.88 (0.62,1.26) |
| Model 4 |  | Reference |  |  | 0.95 (0.62,1.45) |  |  | 0.75 (0.48,1.15) |
| **AS** | 106 (17.8) |  |  | 122 (21.2) |  |  | 244 (22.9) |  |
| Model 1 |  | Reference |  |  | 1.24 (0.93,1.66) |  |  | 1.37 (1.07,1.77) |
| Model 2 |  | Reference |  |  | 1.09 (0.80,1.48) |  |  | 1.01 (0.75,1.34) |
| Model 3 |  | Reference |  |  | 1.11 (0.81,1.52) |  |  | 1.01 (0.75,1.35) |
| Model 4 |  | Reference |  |  | 1.09 (0.77,1.56) |  |  | 0.96 (0.68,1.37) |
| **Carotid artery and Femoral artery** | | | | | | | | |
| **TIMT** | 128 (21.5) |  |  | 140 (24.3) |  |  | 330 (31.0) |  |
| Model 1 |  | Reference |  |  | 1.17 (0.89,1.54) |  |  | 1.64 (1.30,2.08) |
| Model 2 |  | Reference |  |  | 0.96 (0.72,1.30) |  |  | 1.10 (0.84,1.45) |
| Model 3 |  | Reference |  |  | 0.97 (0.72,1.31) |  |  | 1.07 (0.81,1.41) |
| Model 4 |  | Reference |  |  | 1.08 (0.77,1.51) |  |  | 1.18 (0.84,1.65) |
| **Plaque** | 108 (18.1) |  |  | 124 (21.5) |  |  | 251 (23.6) |  |
| Model 1 |  | Reference |  |  | 1.24 (0.93,1.65) |  |  | 1.39 (1.08,1.79) |
| Model 2 |  | Reference |  |  | 1.10 (0.81,1.49) |  |  | 1.05 (0.79,1.40) |
| Model 3 |  | Reference |  |  | 1.13 (0.83,1.54) |  |  | 1.06 (0.80,1.42) |
| Model 4 |  | Reference |  |  | 0.95 (0.67,1.34) |  |  | 0.87 (0.62,1.23) |
| **AS** | 172 (28.9) |  |  | 188 (32.6) |  |  | 411 (38.6) |  |
| Model 1 |  | Reference |  |  | 1.19 (0.93,1.53) |  |  | 1.55 (1.25,1.92) |
| Model 2 |  | Reference |  |  | 1.02 (0.78,1.34) |  |  | 1.12 (0.87,1.44) |
| Model 3 |  | Reference |  |  | 1.03 (0.78,1.36) |  |  | 1.09 (0.84,1.40) |
| Model 4 |  | Reference |  |  | 1.03 (0.75,1.40) |  |  | 1.06 (0.78,1.45) |

Note: TIMT, thickened intima-media thickness; AS, atherosclerosis; CI, confidence interval; OR, odds ratio; SDR, the ratio of systolic and diastolic blood pressure. Model 1 was crude; Model 2 was adjusted for age, sex; Model 3 was adjusted for age, sex, BMI, occupation, education, alcohol drinking, smoking, diabetes, and dyslipidemia; Model 4 was adjusted for age, sex, BMI, occupation, education, alcohol drinking, smoking, diabetes, dyslipidemia, SBP, and treatment of AS.


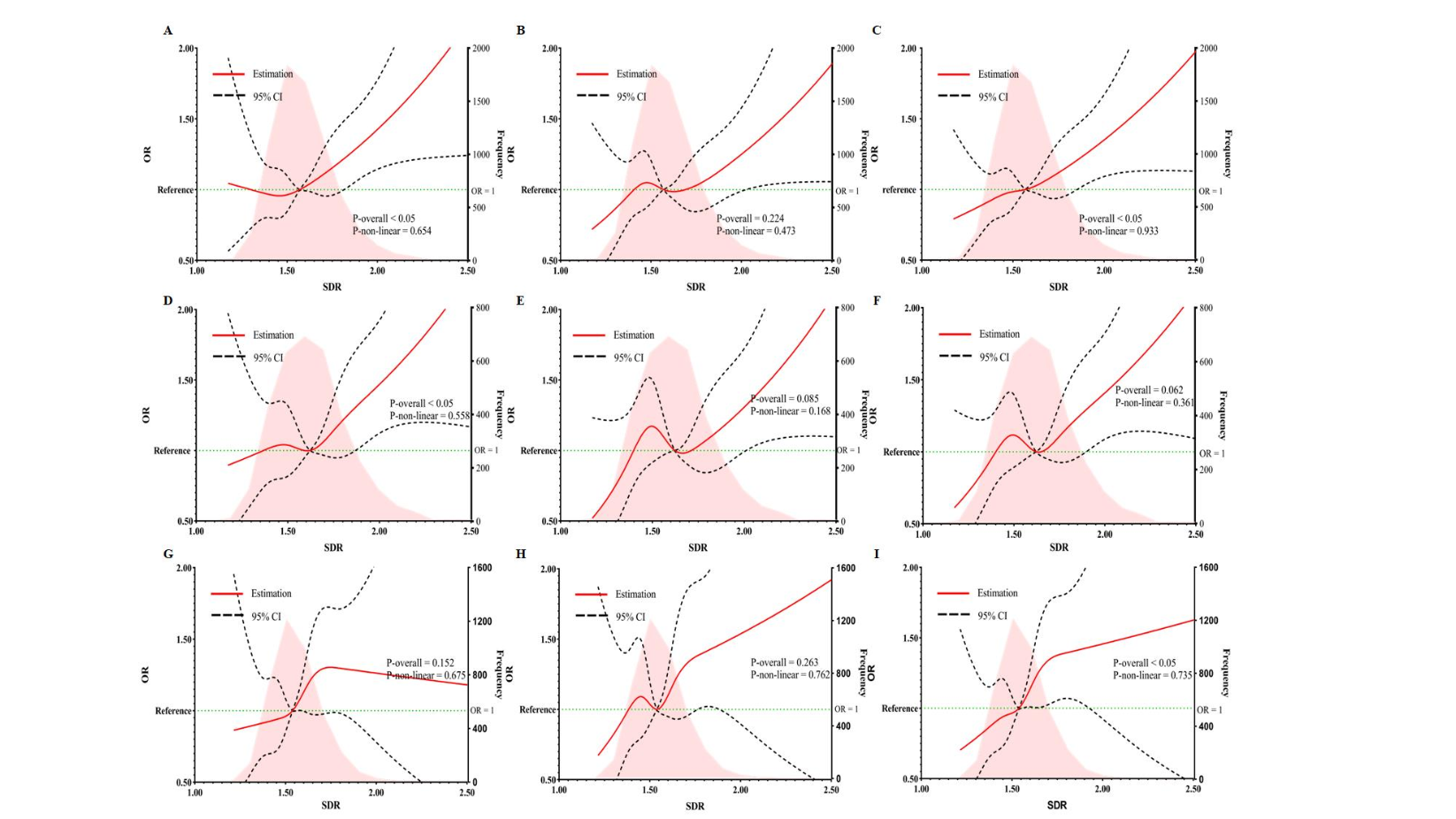


**Supplemental Figure 1. The RCS regression between SDR and the risk of carotid or femoral TIMT (A), plaque (B), and AS (C). The RCS regression between SDR and the risk of carotid or femoral TIMT (D), plaque (E), and AS (F) in hypertension.** **The RCS regression between SDR and the risk of carotid or femoral TIMT (G), plaque (H), and AS (I) in none-hypertension.**

Note: SDR: the ratio of systolic and diastolic blood pressure; OR: odds ratio; CI: confidence interval.


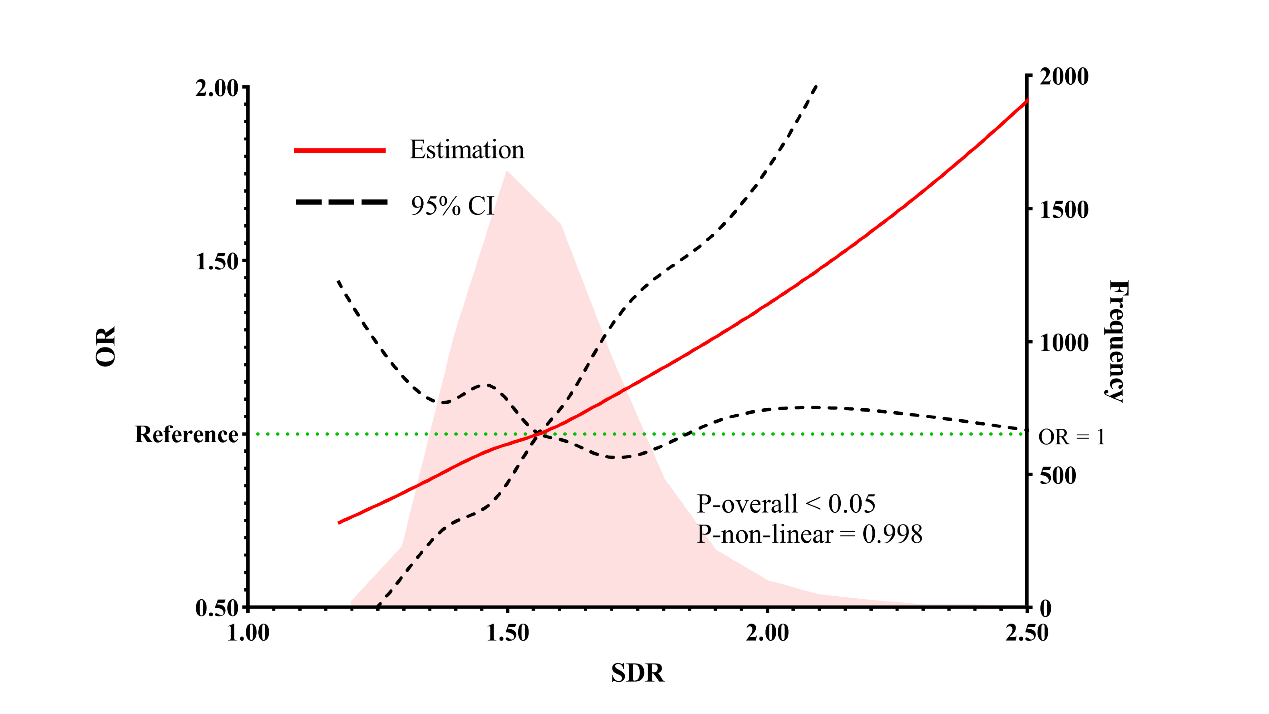


**Supplemental Figure 2. The distribution of SDR and its association with AS in population without antihypertensive medication.**

Note: SDR: the ratio of systolic and diastolic blood pressure; OR, odds ratio; CI, confidence interval.


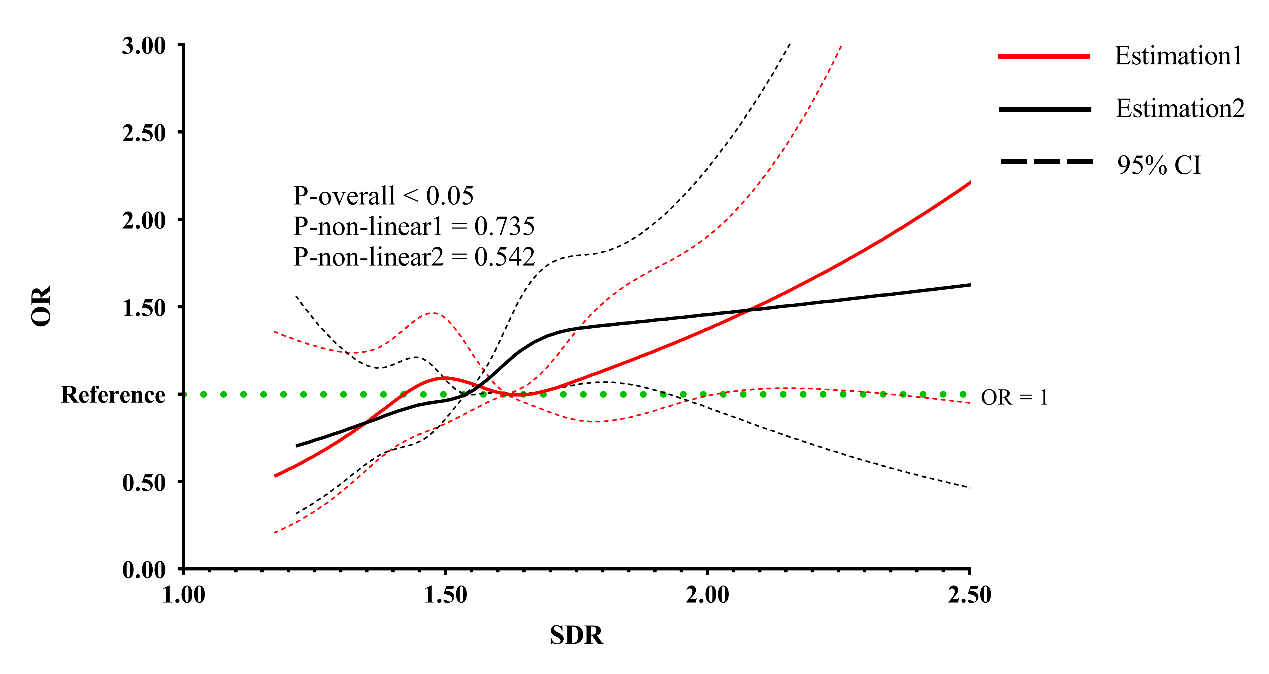
 **Supplemental Figure 3. The potential non-linear association between SDR and AS by hypertension or none -hypertension populations.**

Note: SDR, the ratio of systolic and diastolic blood pressure; OR, odds ratio; CI, confidence interval. Red line represents participants with hypertension. Black line represents participants with none-hypertension.
